# Promising Intervention Approaches to Potentially Resolve Neuroinflammation And Steroid Hormones Alterations in Alzheimer’s Disease and Its Neuropsychiatric Symptoms

**DOI:** 10.14336/AD.2021.0122

**Published:** 2021-08-01

**Authors:** Catia Scassellati, Antonio Carlo Galoforo, Ciro Esposito, Miriam Ciani, Giovanni Ricevuti, Cristian Bonvicini

**Affiliations:** ^1^Biological Psychiatry Unit, IRCCS Istituto Centro San Giovanni di Dio Fatebenefratelli, Brescia, Italy.; ^2^Oxygen-Ozone Therapy Scientific Society (SIOOT), Gorle, Italy.; ^3^University of Pavia, Pavia, Italy.; ^4^Department of Internal Medicine and Therapeutics, University of Pavia, Italy.; ^5^Nephrology and dialysis unit, ICS S. Maugeri SPA SB Hospital, Pavia, Italy.; ^6^P.D. High School in Geriatrics, University of Pavia, Italy.; ^7^Molecular Markers Laboratory, IRCCS Istituto Centro San Giovanni di Dio Fatebenefratelli, Brescia, Italy.; ^8^Department of Drug Sciences, University of Pavia, Italy.; ^9^St. Camillus Medical University, Rome, Italy.

**Keywords:** Neuroinflammation, Steroid hormones, Alzheimer’s Disease, Nutraceuticals, Oxygen-Ozone Therapy, NF-κB, Keap1/Nrf2

## Abstract

Neuroinflammation is a biological process by which the central nervous system responds to stimuli/injuries affecting its homeostasis. So far as this reactive response becomes exacerbated and uncontrolled, it can lead to neurodegeneration, compromising the cognitive and neuropsychiatric domains. Parallelly, modifications in the hypothalamic signaling of neuroprotective hormones linked also to the inflammatory responses of microglia and astrocytes can exacerbate these processes. To complicate the picture, modulations in the gut microbiota (GM) can induce changes in neuroinflammation, altering cognitive and neuropsychiatric functioning. We conducted a web-based search on PubMed. We described studies regarding the cross-talk among microglia and astrocytes in the neuroinflammation processes, along with the role played by the steroid hormones, and how this can reflect on cognitive decline/neurodegeneration, in particular on Alzheimer’s Disease (AD) and its neuropsychiatric manifestations. We propose and support the huge literature showing the potentiality of complementary/alternative therapeutic approaches (nutraceuticals) targeting the sustained inflammatory response, the dysregulation of hypothalamic system and the GM composition. NF-κB and Keap1/Nrf2 are the main molecular targets on which a list of nutraceuticals can modulate the altered processes. Since there are some limitations, we propose a new intervention natural treatment in terms of Oxygen-ozone (O_2_-O_3_) therapy that could be potentially used for AD pathology. Through a meta-analytic approach, we found a significant modulation of O_3_ on inflammation-NF-κB/NLRP3 inflammasome/Toll-Like Receptor 4 (TLR4)/Interleukin IL-17α signalling, reducing mRNA (p<0.00001 Odd Ratio (OR)=-5.25 95% CI:-7.04/-3.46) and protein (p<0.00001 OR=-4.85 95%CI:-6.89/-2.81) levels, as well as on Keap1/Nrf2 pathway. Through anti-inflammatory, immune, and steroid hormones modulation and anti-microbial activities, O_3_ at mild therapeutic concentrations potentiated with nutraceuticals and GM regulators could determine combinatorial effects impacting on cognitive and neurodegenerative domains, neuroinflammation and neuroendocrine signalling, directly or indirectly through the mediation of GM.

Neuroinflammation is a defence mechanism aimed to protect the central nervous system (CNS) in response to a variety of insults, including infection, traumatic injury, toxic metabolites, or autoimmune events [1]. While an acute neuroinflammatory response is generally gainful to the CNS, minimizing further injury and contributing to the tissue homeostasis, chronic neuroinflammation can give rise to severe damage to the neuronal compartment, interfering with its homeostatic integrity, ruffling the balance between reparative responses and pro-inflammatory events [2].

Glial cells are the critical executors involved in these mechanisms. In [3], the authors describe, as a well-orchestrated symphony, the cross-talks between various groups of these glial cells in CNS neuroinflammation and that they define an extremely complex and dynamic process. Glial cells are strongly implicated in the immune response inside the brain, regulate its homeostasis, modulate synaptic activity, neural regeneration, influence its development, and have a control on endocrine system [4]. They enclosed microglia and macroglia. Microglia are macrophage-like cells that regulate the inflammatory response of the neural tissue to injury or infection. They always act as the first violin or play the sonata part of the beginning movement of the CNS inflammation symphonies [3, 5]. Macroglia are subdivided in four specialized cell types: ependymal cells, Schwann cells, oligodendroglia, and astroglia. All them are grouped as astroglia because they have in common the expression of glial fibrillary acidic protein (GFAP). Astrocytes are the most abundant cell type in the CNS. As compared to microglia, their responses to inflammatory stimuli appear to be more passive, but they are also considered to exhibit active parts in the initiation of inflammation [6, 7]. Indeed, whereas microglia participate in the first line of defence and orchestrate neuroinflammation by sending immunosignals to astrocytes, astrocytes amplify and propagate the signals received. In addition, astrocytes are known to control the metabolism of neurons, because they have close contacts with neurons and capillary vessels [3].

Hormones and the neuroendocrine system are additional actors strongly interrelated with microglia-astrocytes cross-talk. Circulating steroids produced by the adrenal glands, the gonads and the placenta readily cross the blood brain barrier (BBB) and reach their target cells in the CNS by diffusion; these are termed ‘neuroactive’ steroids [8-10]. In addition, the CNS can synthesize steroids *de novo* [8, 11]. Steroids produced in the CNS and circulation-derived steroids have indistinguishable effects in the CNS [12]. Hormonal actions in the brain are exerted equally on neurons and glial cells, and the ability of glia to both respond to and produce hormones has profound functional implications in the neuroendocrine system functions. Steroid hormones released during stressful conditions (activation of Hypothalamic Pituitary Adrenal, HPA axis) influence processes not only linked to reproduction, sexual differentiation and behaviour, but also to brain development, cognition, memory, synaptic plasticity, neurogenesis, brain homeostasis, and the central immune response [4, 5]. Glial cells express many of the same hormone receptors expressed on neurons: receptors for melatonin, for thyroid and steroid hormones, vasopressin, oxytocin, leptin, corticotropin-releasing factor, glucagon, insulin, and Insulin Growth Factor-1 (IGF-1).

Neuroinflammation is being increasingly recognized as a potential mediator of cognitive impairments [13]. The impact of age on neuroinflammatory responses including glial activation, increased production of proinflammatory cytokines, and aberrant neuronal signalling could magnify the deterioration of the CNS microenvironment in neurodegenerative diseases (NDs), contributing to accelerate cognitive decline. NDs such as Alzheimer's Disease (AD) are characterized by memory impairment and by general decrease in both cognitive functions and daily living competences, that lead to a complete loss of autonomy. These functions are strongly linked to a high level of chronic neuroinflammation, attributed to the activation of microglia cells and to the release of numerous pro-inflammatory cytokines [14]. These processes can have a crucial impact also on neuropsychiatric symptoms often associated to AD pathology [15].

AD is one of the most prevalent NDs manifesting 45 million people worldwide. Deposition of protein aggregates, extracellular amyloid plaques (Aβ), intracellular tau (τ) or neurofibrillary tangles (NFT), and loss of synaptic connections in specific regions of brain are the main features observed in AD [16-18]. This accumulation of neurotoxic Aβ oligomer peptides along with τ protein mediates neurodegeneration, thus causing neuroinflammation, neurotransmitter imbalance, oxidative stress, impairment in synaptic connection, cholinergic denervation, neuronal loss, and dendritic alterations [19].

While the presence of cognitive impairment is necessary and sufficient for a diagnosis of AD, associated neuropsychiatric symptoms (known collectively as Behavioral and Psychological Symptoms of Dementia, or BPSD) are prevalent and can significantly impact the prognosis and management of AD [20]. BPSD include emotional, cognitive/perceptual, and behavioural disturbances that are similar to those observed in psychiatric disorders [20]. For instance, in our AD Italian cohort, we detected 52% of AD patients showing higher severity in agitation symptomatology, 48% in irritability, 42% in night-time behaviour disturbances and, finally, 39% in aberrant motor behaviour. Slightly less representative, there were: apathy (28% higher severity), delusions (29% higher severity), anxiety (25% higher severity), depression (22% higher severity), and hallucinations (21% higher severity) [21].

Changes in the hypothalamic signalling of neuroprotective hormones, which also regulate the inflammatory responses of microglia and astrocytes, accelerate dysfunctions in NDs, including AD.

The use of complementary/alternative therapeutic approaches targeting the sustained inflammatory response and the dysregulation of hypothalamic dysfunction could represent good strategies to overcome the limitations linked to the actual pharmacological treatments for AD. Interestingly, it has been reported that alterations in the gut microbiota (GM) (intestinal flora) can induce changes in neuroinflammation, altering cognitive [22] and neuropsychiatric functioning [23], and that also can raise the possibility of therapeutic intervention through the manipulation of the GM, to overcome the outcomes of these pathologies. A literature research (see supplementary Table 1) on topic “natural compounds (nutraceuticals/phytochemicals)” and “Alzheimer’s Disease” evidenced over 119 reviews (Table 1S), only considering the last two years (2019-2020). This suggests the huge attention given to these types of approaches that could potentially resolve neuroinflammation/neuro-degeneration processes in AD.

In this review, we want to: 1. evidence the role of microglia-astrocytes cross-talk on the molecular mechanisms of neuroinflammation in patho-physiological conditions; 2. highlight the interactions between steroid hormones and microglia-astrocytes system in patho-physiological conditions; 3. describe a literature overview of the recent development of adjunct therapeutic options in terms of natural compounds (nutraceuticals/phytochemicals) that potentially could resolve neuroinflammation, steroid hormones alteration and GM composition, that in turn can influence the neurodegeneration in AD. The transcriptional factors NF-κB (Nuclear factor kappa light chain enhancer of activated B cells) and Keap1/Nrf2 (Kelch-like ECH-associated protein 1/Nuclear factor E2-related factor 2) are the main molecular targets on which a list of nutraceuticals can modulate the altered processes. We discussed the state of art on clinical trials, nutraceuticals, and AD. Since there are some limitations linked to the use of these compounds, we suggest new therapeutic interventions that could be applied for this pathology. We showed the rationale that supported the efficacy of the Oxygen (O_2_)-Ozone (O_3_) therapy on different pathological conditions, including degenerative disorders such as multiple sclerosis [24, 25]. This therapy could represent a new therapeutic intervention for AD, due the widely demonstrated properties of O_3_ as anti-inflammatory/immune and steroids stimulator and anti-microbial activity against virus, bacteria, fungi [26, 27]. We presented meta-analyses of the modulation of O_3_ on the inflammatory NF-κB/inflammasome NLRP3/Toll-Like Receptor 4 (TLR4)/Interleukin-IL-17α signaling.

## Microglia-astrocytes cross-talk: biological mechanisms in physiological conditions

Microglia and astrocytes are cells of profoundly different origin and with distinct functions in the CNS. They play indispensable instructive roles in neurodevelopmental processes, such as gliogenesis, neurogenesis, axonal outgrowth, synaptogenesis, angiogenesis, and synaptic pruning [28].

An exhaustive recent review [29] illustrates how a profound cross-talk between microglia and astrocytes can produce microglia- and astrocyte-derived signals, as functional determinants for the fates of astrocytes and microglia, respectively. By releasing diverse signalling molecules, both microglia and astrocytes establish autocrine feedback and their bidirectional conversation for a tight reciprocal modulation during CNS insult or injury. Microglia, the constant sensors of changes in the CNS microenvironment and restorers of tissue homeostasis, serve as the primary immune cells of the CNS and, at the same time, regulate the innate immune functions of astrocytes. Moreover, microglia determine the functions of reactive astrocytes, ranging from neuroprotective to neurotoxic. Conversely, astrocytes through their secreted molecules regulate microglial phenotypes and functions ranging from motility to phagocytosis. Altogether, microglia-astrocytes cross-talk contributes to physiology and pathology of the CNS [29-31].

Microglia encompass a set of highly active and versatile cells, traditionally considered the immune cells of the CNS [32]. These macrophage-like cells originating from macrophages invade the brain during early development and are ubiquitous in the CNS. In the normal, uninjured brain, they are referred to as “resting microglial” and serve as highly active sensors of the biochemical or bioelectric microenvironment [33], determining synaptic pruning and production of cytokines and neurotrophic factors [34]. To play these functions, microglial cells are supplied of receptors for neurotransmitters, neuropeptides, hormones, immune signals, and other molecules that allow the microglia to efficiently scan their territory [35].

Astrocytes are the most abundant and versatile cells in the brain, and they are involved in a diversity of functions in the CNS. These roles include: support of neurogenesis and gliogenesis; regulation of synaptogenesis, cerebral microcirculation, extracellular ion concentrations and extracellular pH, and brain water homeostasis; guidance in neuronal migration; provision of energy substrates for neurons; modulation of neurotransmitter signalling and recycling, synaptic transmission, and neuroendocrine functions; production of cytokine and neurotrophins; integration and regulation of synaptic networks [4, 34, 36].

As illustrated in [3], the progression of neuroinflammation is a balancing process of two counteracting forces (pro- and anti-inflammation). The heterogeneity of glia cells and the diverse mechanisms of intercellular regulation shape the “two-sided” character of neuroinflammation. The outcome of neuroinflammation depends on which side is winning in this tug of war. Activated glia cells develop into two classes of counteracting phenotypes in the progression of neuroinflammation: 1. the pro-inflammatory microglia (M1 microglia, M1-like phenotype, classical activation) secrete pro-inflammatory mediators (Tumor Necrosis Factor-γ, TNF-γ; TNF-α; IL-1β; IL-6; IL-18; IL-23; Interferon-γ, IFN-γ), as well as Reactive Oxygen Species (ROS), nitric oxide (NO), and other immunomodulatory factors causing inflammatory response at the site of injury [37]. Cytokines (IL-1α/β, TNF-α, complement component C1q) secreted by activated microglia change astrocytes into a pro-inflammatory phenotype, secreting in turn CCL2 (C-C-motif chemokine ligand 2), CX3CL1 (C-X3-C Motif Chemokine Ligand 1), CXCL10 (C-X-C motif chemokine ligand 10), GM-CSF (granulocyte-macrophage colony-stimulating factor), and IL-1, which activate the pro-inflammatory microglia. This contributes to the death of neurons and oligodendrocytes. This system is strongly linked to NF-ĸB signalling [19]. 2. The protective microglia (M2 microglia, M2-like phenotype, alternative activation) secrete anti-inflammatory mediators (IL-4; IL-10; Transforming Growth Factor-β, TGF-β; IGF-1; Vascular endothelial growth factor, VEGF; Brain-derived neurotrophic factor, BDNF; Platelet-derived growth factor, PDGF) and participate in cellular debris clearance and tissue repair [38-41]. M2 anti-inflammatory phenotype is classified as M2a, M2b, and M2c [42]. M2a is related to repair and regeneration, M2b is a transitional state involved in immune response, whereas M2c is implicated in neuroprotection and release of anti-inflammatory cytokines [43].

A complex regulatory loop has been demonstrated that involved two crucial nuclear transcription factors in the balancing pro/anti-inflammatory progression: not only NF-ĸB, but also Keap1/Nrf2 [44]. The Keap1/Nrf2/ARE (antioxidant responsive element) signalling pathway regulates anti-inflammatory genes expression and inhibits the progression of inflammation directly by HO-1 (heme oxygenase-1) and indirectly by CO (carbon monoxide) proteins. On the other hand, IκB kinase-dependent (IKK-dependent) phosphorylates NF-ĸB, leading to its translocation into the nucleus and activation of proinflammatory cytokines. Thus, in presence of stimuli (e.i oxidative stress), the Nrf2 pathway inhibits NF-ĸB activation by preventing the degradation of IĸB-α, and increases HO-1 expression, leading to activation of anti-inflammatory system. Moreover, some pro-inflammatory products induced by NF-ĸB activation such as COX2 (cyclooxygenase 2) can act as inducers of Nrf2, that ultimately leads to the suppression of oxidative stress. On the other hand, NF-ĸB-mediated transcription reduces Nrf2 activation by decreasing ARE genes transcription because free CREB binding protein competes with Nrf2 for CBP (cAMP-response-element binding protein). Moreover, NF-ĸB increases the recruitment of histone deacetylase (HDAC3) to the ARE region and hence Nrf2 transcriptional activation is prevented.

If an acute inflammatory reaction favours tissue healing and helps restoring CNS homeostasis [45], on the other hand excessive glial activation causes neuronal loss, which, in turn, establishes a state of pernicious chronic neuroinflammation [45, 46], exacerbated by the failure to maintain the homeostasis of the CNS [3]. This chronic inflammation is one of the main aetiopathological mechanisms in NDs such as AD.

## Microglia-astrocytes cross-talk: biological mechanisms in pathological conditions (Alzheimer’s Disease)

**Figure 1.**
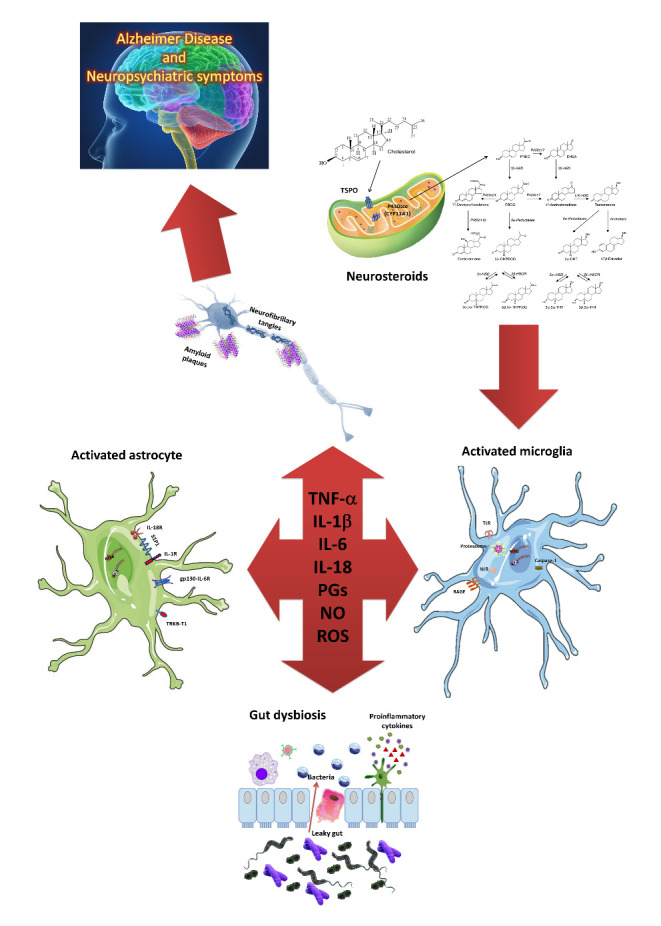
Neuroinflammation, steroid hormones alteration and gut microbiota (GM) modulation in Alzheimer Disease (AD) and its neuropsychiatric symptoms. We illustrate three crucial features underlying the neurodegenerative disorders such as AD and its neuropsychiatric manifestations (Behavioral and psychological symptoms of dementia, BPSD): a) chronic neuroinflammation and the complex interplay between astroglia and microglia, b) alterations in the hypothalamic signalling of neuroprotective hormones linked also to the inflammatory responses of microglia and astrocytes, and c) modulations in the GM, inducing changes in brain activities contributing to neuroinflammation, and altering cognitive and neuropsychiatric functioning. Tumour necrosis factor-α (TNF-α); Interleukin (IL)-1β; IL-6; prostaglandins (PGs); nitric oxide (NO); Reactive oxygen species (ROS); Toll-like receptors (TLRs); Nuclear factor Nf-κB; Activator protein 1 (AP-1); Receptor for advanced glycation endproducts (RAGE); Glycoprotein 130 (gp130); Neurotrophic Receptor Tyrosine Kinase 2 (TRKB-T1); Smad interacting protein 1 (SIP1); Translocator protein (TSPO); PREG (Pregnenolone); Dehydroepiandrosterone (DHEA); Progesterone (PROG).

Amyloid precursor protein (APP) is cleaved producing Aβ oligomer peptides that aggregate [36]. As reported in [12, 19], Aβ peptides work as danger signals, activating microglia through Pattern Recognition Receptors (PRRs), such as TLR4, Receptor for Advanced Glycation End products (RAGE) and NOD-like receptors (NLR). These activations change the transcriptional program of the microglia cells, inducing expression of transcription factors such as NF-κB and AP-1 (Activator Protein 1), which, in turn trigger the production of proinflammatory cytokines (TNF, IL-1β, IL-6), prostaglandins, NO and ROS [47, 48]. A feature of activated microglia is the inflammasome NLRP3, which is a protein complex mediating the activation of caspase 1-dependent cleavage and the release of IL-1β and IL-18 [49]. Recent studies support a crucial TLR4 cross-talk with NLRP3 in AD pathology [50]. Inflammatory conditions may induce APP and secretase expression, leading to Aβ aggregation and τ kinase activation, resulting in NFT formation [48, 51, 52]. In addition, microglia activate astrocytes through secretion of TNF and IL-1β [47], and, in turn pro-inflammatory cytokines (TNF, IL-1β, IL-6) may induce neuronal (cholinergic, glutamatergic, GABA-ergic neurons) apoptosis [47, 53-59]. Apoptosis of neurons results in release of ATP (Adenosine Triphosphate), which further activates microglia through purinergic P2X7 receptor, entering in an auto-stimulatory loop inducing T-cell infiltration [36]. Neuroinflammation and neurodegeneration create thus a vicious cycle driving disease progression (Fig. 1). Some studies found higher TNF-α and lower TNF-β, an anti-inflammatory cytokine levels in the cerebrospinal fluid (CSF) of mild cognitive impairment (MCI) patients who progressed to AD, compared with the control subjects. Some cytokines including IL-1β, IL-6, and TNF-α have slowly increased levels from the early stage of the disease, while the levels of other cytokines including IL-18, MCP-1 (Monocyte chemoattractant protein-1), and CXCL10/IP-10 (IFN-γ inducible protein) can peak at a certain stage of the disease [19].

Interestingly, this chronic neuroinflammation is also associated to neuropsychiatric symptoms [60, 61]. For instance, in serum of mild to severe AD patients, an increase of TNF-α levels was found in apathy, anxiety, depression and agitation symptoms in AD [62, 63]. Holmgren *et al*. [60] found, in CSF of patients with dementia, correlations between the levels of the soluble IL-1 receptor type II (sIL-1RII) with apathy symptom, and IL-6 with anxiety. Overexpression of IL-1β and administration of Lipopolysaccharide (LPS) or endotoxins (agents that promote the activation of microglia) have shown to exacerbate the τ accumulation in AD mice, triggering a depressive symptomatology [64-66].

Recently, it has been hypothesized that the chronic neuroinflammation observed in AD is associated with an alteration of the balance of GM (dysbiosis), which can negatively affect neuronal activity [67]. GM modulates important processes, such as microglia maturation and activation, neurogenesis, myelination, synaptic pruning, and BBB permeability. The GM-brain axis links GM and the brain via metabolic, endocrine, neural, and immune pathways that are crucial for the maintenance of brain homeostasis [68]. Emerging evidence indicate that gut dysbiosis may aggravate Aβ aggregation and neuroinflammation in the development of AD [69]. Gut dysbiosis induces the decrease of beneficial substances (such as Short-chain Fatty Acids, SCFAs and Hydrogen, H_2_) and the increase of harmful substances (such as amyloids, bile acids and Trimethylamine N-Oxide, TMAO). In particular, SCFAs (acetate, butyrate, lactate, propionate) are main metabolites of the fermentation of dietary fibers by the GM [70], and are the major players in the maintenance of gut and immune homeostasis with anti-inflammatory, antitumorigenic, and antimicrobial properties [71]. SCFAs also have roles in the inhibition of NF-κB, histone deacetylation, and in the activation of G protein-coupled receptors [72]. This disequilibrium causes the reduction in permeability of both intestinal mucosal barrier and BBB, activating peripheral immune responses, and increasing peripheral and central oxidative stress levels.

This is reflected on the AD pathology progression by increasing amyloid plaque formation and accumulation, neuroinflammation, stress granules and insulin resistance [69] (Fig. 1).

Nine studies to date summarized in [69], evidenced alterations of GM composition in AD models. For instance, Friedland, 2015 [73] reported that AD patients showed higher profile of gram-negative bacteria, which cause a disruption of the mucosal barrier, as well as produce amyloid β-bacteria (*Bacillus subtilis, Klebsiella pneumonia, Mycobacterium spp., Streptococcus spp*.). Anti-inflammatory taxa (i.e., *Eubacterium rectale*) were less abundant in the AD patients’ feces, whereas pro-inflammatory taxa (i.e., *Escherichia* and *Shigella*) that were linked to pro-inflammatory cytokines and amyloid deposition in the brain, were more common [74]. Restoring and remodelling GM composition thus may result in a strategic breakthrough in the treatment approaches, especially with a view to preventing AD [69].

## Steroid hormones and neuroinflammation: biological mechanisms in physiological conditions

Steroid hormones (sex hormones and glucocorticoids, GCs) have been demonstrated to play a role in different cellular processes in the CNS, ranging from neurodevelopment to neurodegeneration, as these hormones target all neuronal cells (neurons, glia, and/or immune cells) [75]. Their functions regard behaviour, cognition, memory, learning, and motor activities [76].

Steroid hormones are a family of mediators derived from the common precursor cholesterol that includes GCs (involved in stress responses), mineralocorticoids (MNs, which control osmotic balance), androgens, estrogens and progestagens [77]. GCs have been used for more than 50 years in the treatment of several diseases with an in?ammatory component [78]. Sex hormones are also thought to play a signiﬁcant modulatory role, contributing to sexual dimorphism in the incidence of autoimmune and in?ammatory diseases [75].

As reported in [5], the integration of hormonal signalling by glial cells occurs in at least two fundamental ways: 1) the hormone acts directly on the glia through specific receptors, which in turn signal to the neuron to modulate its function. Signalling to the neuron may involve secretion of a growth factor, neurohormone, or transmitter-like substance [79]; and 2) the steroids act first on neurons, to initiate a circular conversation between neurons and astrocytes that ultimately returns to neurons.

As described in [12], 17β-estradiol can activate estrogenic receptors ERα and ERβ present on microglia. In this way, it mediates anti-inflammatory effects by suppressing TNF, IL-6 or iNOS (inducible Nitric oxide Synthase) expression. Additionally, through the membrane receptor GPR30 (G protein-coupled receptor 30), 17β-estradiol can stimulate rapid signalling events, such as calcium (Ca^2+^), cAMP or ERK signalling, potentially mediating anti-inflammatory effects. 17β-estradiol was also reported to negatively regulate NLRP3 inflammasome activation. In astrocytes, it can modulate ERα signalling, decreasing chemokines expression.

In the CNS, progesterone and allopregnanolone derive from the periphery, the astrocytes, and/or the oligodendrocytes. In microglia, progesterone activates its receptors: membrane PRα (progesterone receptor α) and PGMRC1 (progesterone membrane receptor component 1). In this way, it can down-regulate the NLRP3 inflammasome activation, inhibit the expression of pro-inflammatory genes (*TNF; IL-6; iNOS; Major Histocompatibility Complex class II, MHCII; COX2*), and enhance the TREM2 (Triggering receptor expressed on myeloid cells 2), CD206 (Cluster of Differentiation 206) or TGFβ expression. Similarly, the activation of PRα and PGMRC1 receptors expressed on astrocytes allows progesterone to induce BDNF release in a Pgrmc1-dependent manner, thereby promoting cell survival. On the other hand, allopregnanolone binds to GABA-A receptors in microglia as well as in astrocytes mediating anti-inflammatory effects through NF-κB inhibition [12].

DHEA (Dehydroepiandrosterone) is synthesized in astrocytes and neurons, while it also derives from the circulation. In microglia, it exerts anti-inflammatory effects through activation of the NGF (Nerve Growth Factor) receptor TrkA, which triggers the AKT/CREB signalling cascade, leading to an enhanced *Jmjd3 (Jumonji Domain Containing 3)* gene expression. Moreover, it can be metabolized to ADIOL (5-androstene-3 beta), which binding to ERβ, regulates negatively pro-inflammatory genes expression. Through the regulation of cytokines and NO secretion, it affects astrocytes activation and neuronal survival [12].

## Steroid hormones and neuroinflammation: biological mechanisms in pathological conditions (Alzheimer’s Disease)

In AD, changes in the hypothalamic signalling of neuroprotective hormones, which also regulate the inflammatory responses of microglia and astrocytes, can further accelerate hypothalamic dysfunction [34].

In neurons, stressors such as infections or AD-related genetic and environmental factors or secretion of DAMPS (damage-associated molecular patterns) would put immune system on alert and possibly also stimulate HPA axis. A persistent activation of HPA axis can result in loss of its regulation, and at the same time in chronically high GCs levels and glucorticoids receptors (GR) dysfunction in immune cells. Microglia and astroglia remain activated creating a pro-inflammatory environment (activation of NF-κB pathway, IL-1β, IL-6, TNF-α) and augmenting oxidative stress [75]. The uncontrolled activation of NF-κB signalling by microglia, and the resulting alterations in other glial cell types and neurons, impair the hypothalamic regulation of hormonal secretion, as well as the hypothalamic ability to sense and respond to the hormonal feed-back received from the endocrine glands. This creates a vicious circle in which the aging-associated decrease in anti-inflammatory and neuroprotective hormone levels as well as their hypothalamic signalling further impairs microglia and hypothalamic function, generating a cascade of events, resulting in the inability of the brain to properly control body homeostasis [34]. Disruption in BBB resulting in T cell infiltration further promotes glial activation (Fig. 1).

Different lines of evidence showed that AD can significantly alter neurosteroidogenesis [12] and substantial changes in their levels have been observed in the AD brain. As summarized in [12, 80], allopregnanolone was found to be significantly decreased in the prefrontal and temporal cortices of AD patients [81-83]. A general trend toward decreased levels of progesterone was observed in all AD patients' brain regions (frontal cortex, striatum, hypothalamus and hippocampus) compared with controls [84]. DHEA and DHEAS were detected to be significantly lower in aged AD patients than age-matched nondemented controls, especially in the striatum, cerebellum, frontal cortex, amygdala, hippocampus, and hypothalamus, and negatively correlated with high levels of cortical Aβ and phosphorylated τ proteins [17, 81-89]. Interestingly, DHEAS or estrogens administration was shown to improve cognitive performance in different AD animal models [12]. Changes in sex steroid levels are also relevant to AD [80]. Women with AD aged 80 years and older exhibited significantly lower brain 17β-estradiol than age-matched nondemented controls [90, 91]. In another study, 17β-estradiol levels were significantly increased in the prefrontal cortex and in the hippocampus after bilateral infusion of Aβ into the male rat hippocampus [92].

## Potential adjunct therapeutic options targeting neuroinflammation/neurodegeneration in AD: clinical trials

**Figure 2.**
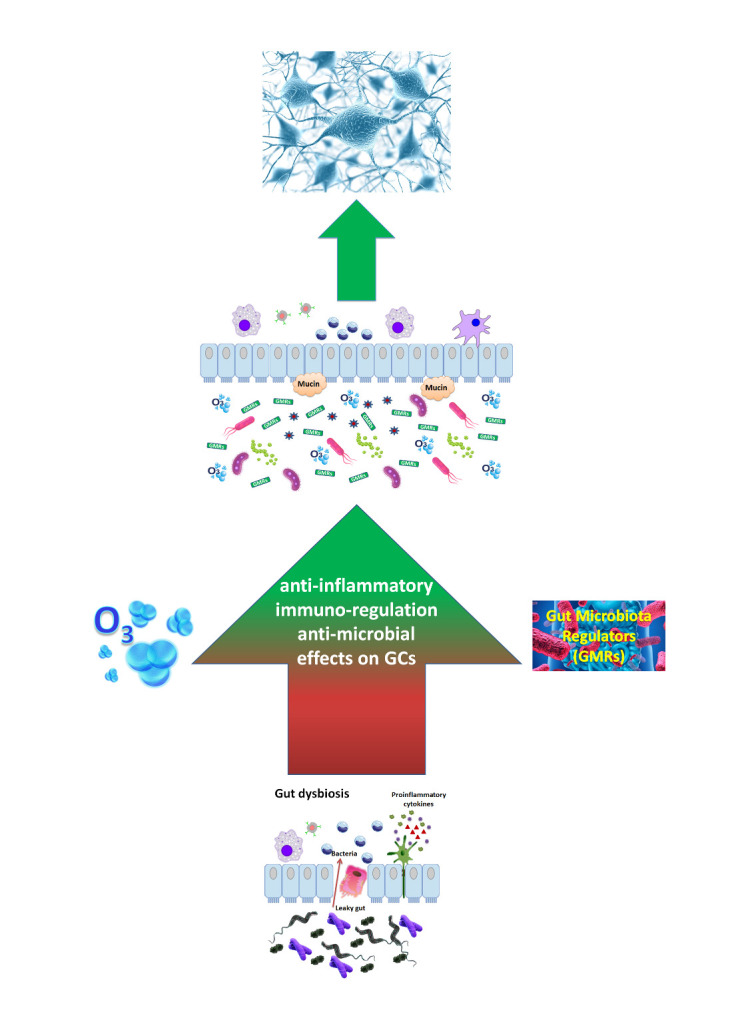
The potential positive effects of Oxygen-Ozone O_2_-O_3_ therapy and Gut Microbiota Regulators (GRMs) on gut microbiota (GM) and consequently on neuroinflammation and neuroendocrine system. We illustrate how a natural bio molecule such as Ozone (O_3_) showing immune, anti-inflammatory, anti-microbial properties with effects on glucocorticoids (GCs), could represent a new treatment to delay neuroinflammation/neurodegeneration in Alzheimer Disease (AD), where the balance between pro-anti-inflammatory system and the hypothalamic signalling and GM composition are impaired. Moreover, we illustrate that the supplementation of GRMs in terms of Probiotics and GV-971, already used for AD, can potentiate the O_3_ activities, determining combinatorial effects that could impact on cognitive and neuropsychiatric domains, neuroinflammation and hypothalamic signalling, directly or indirectly through the mediation of GM.

As most of the drugs have failed in finding a medical solution, natural products emerge as a viable also preventive therapeutics' pathway. Considering that AD is a multifactorial disease, phenolic compounds, alkaloids, terpene/terpenoids, carotenoids, sulfur-compounds, as well as some other plant-derived miscellaneous compounds offer the advantage of a multitarget approach, tagging different molecular sites in the human brain, as compared with the single-target activity of most of the drugs used for AD treatment. Here, we want to underline that they modulate several dysregulated mediators, especially those with a near interconnection with NF-κB-Keap1/Nrf2 signalling: attenuating NF-κB and potentiating Keap1/Nrf2, these natural compounds could play a pivotal role in combating AD. From over 119 reviews published in 2019-2020 (supplementary Table 1), there are works where the authors reported the biological molecular effects of nutraceuticals on NF-κB [93-95], Keap1/Nrf2 [96-98] as well as steroid hormones system that we summarized in Table 1. We also presented evidence on the implication that these natural compounds show on neurodegeneration in terms of Aβ and τ toxicity and aggregation. Here, we described the nutraceuticals mostly implicated in clinical trials for AD. In Figure 2, we illustrated the mechanisms of action of GM regulators (Probiotics and Sodium oligomannate, GV-971, Table 1) on neuroinflammation/neurodegeneration.

**Figure 3.**
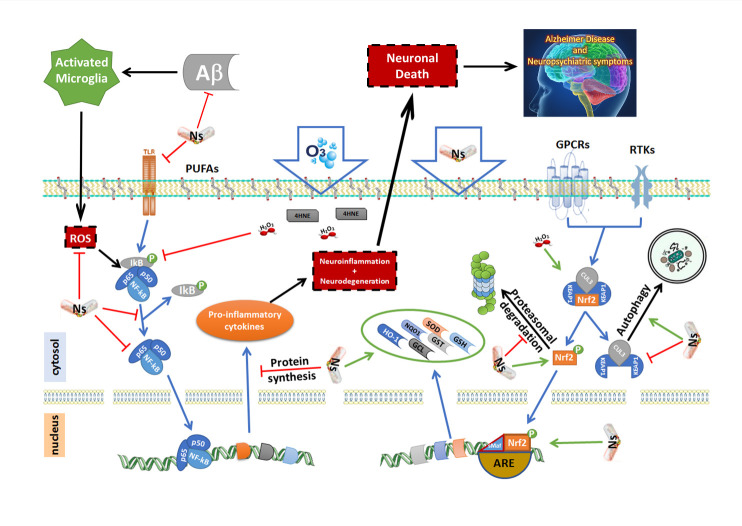
Mechanisms of action of Nutraceuticals (Ns) and Ozone (O_3_) on NF-kB (nuclear factor kappa-light-chain-enhancer of activated B cells)/Keap1 (kelch-like ECH-associated protein)/Nrf2 (nuclear factor erythroid 2-related factor 2) molecular pathways. Here, we illustrate how NF-κB and Keap1/Nrf2 signalling pathways involved in the neuroinflammation/neurodegeneration in Alzheimer’s Disease mechanisms can be therapeutic targets for Nutraceuticals (Ns) and Ozone (O_3_) molecule. In the absence of stimuli, NF-κB is found in cytoplasm bound to the inhibitory IκB (nuclear factor of kappa light polypeptide gene enhancer in B-cell inhibitor) proteins. In response to stimuli (Reactive oxygen species, ROS), IκB proteins are rapidly phosphorylated by IκB kinase and ultimately degraded by the 26S proteasome. The resulting release of NF-κB and subsequent translocation to the nucleus promote its action on target genes involved in inflammation/immunity (pro-inflammatory cytokines). If chronic, this can provoke neuroinflammation/neurodegeneration. O_3_ (by second messengers: Hydrogen Peroxide, H_2_O_2_ and 4-Hydroxynonenal, 4HNE) and Ns can block this system, contributing to an anti-inflammatory response. On the other hand, the Keap1/Nrf2 pathway is organized in this way: in the absence of stimuli, Nrf2 binds to its repressor Keap1, an adapter between Nrf2 and Cullin 3 protein, which leads to ubiquitination followed by proteasome degradation. Under oxidative stress, after heterodimerization with the Muscoloaponeurotic Fibrosarcoma (MAFs), the Nrf2 translocates to the nucleus, where it dimerizes and binds to ARE (Antioxidant Response Element) genes: *Superoxide dismutase (SOD), Glutathione (GSH), Glutathione-S-Transferase (GST), Glutamate-cysteine ligase (GCL), heme oxygenase 1 (HO-1), NADPH:quinone oxidoreductase 1 (NQO1)*, and other anti-oxidant enzymes, provoking a strong anti-oxidant response. *HO-1* is a gene encoding enzyme that catalyses the degradation of heme in carbon monoxide (CO), that in turn is an inhibitor of the NF-κB pathway, thereby decreasing the expression of pro-inflammatory cytokines. HO-1 directly inhibits the pro-inflammatory cytokines and activates the anti-inflammatory cytokines. Keap1 can also prevents NF-κB activity *via* IKK (IκB kinase) inhibition. O_3_ (by second messengers: Hydrogen Peroxide, H_2_O_2_ and 4-Hydroxynonenal, 4HNE) and Ns can activate Keap1/Nrf2 signalling exercising both anti-inflammatory and anti-oxidant effects. O_3_ and Ns can activate pro-autophagy and anti-apoptosis mechanisms and block proteosome degradation. Ns can have significant effects on Aβ and τ toxicity and aggregation, decreasing the neurodegeneration.

Figure 3 represents the mechanisms of action of the nutraceuticals reported in Table 1 and described below, specifically on NF-κB and/or Keap1/Nrf2 signalling pathways.

**Table 1:** Anti-inflammatory/immunostimulation activities of Natural Compounds on NF-κB (nuclear factor kappa-light-chain-enhancer of activated B cells)/Keap1 (kelch-like ECH-associated protein)/Nrf2 (nuclear factor erythroid 2-related factor 2) pathways in Alzheimer’s Disease.

Natural Compounds			Clinical Trials	References
Polyphenols	•Anthocyanin, •Apigenin, •Chalcones, •Cinnamon, •Curcumin, •Gallic acid, •Genistein, •Epigallocatechin gallate, •Lycopene, •Luteolin, •Kaempferol, •Macranthoin G, •Naringin,	•Oleuropein aglycone, •Pinocembrin, •Pterostilbene, •Resveratrol, •Quercetin, •Naringenin, •Obovatol, •Rutin, •Punicalagin, •Salidroside, •Xanthohumol, •4-O-methylhonokiol.	Promising (Curcumin). Promising (Epigallocatechin Gallate). One trial (Genistein). Promising, best efficacy for BPSD (Ginkgo). Promising (Resveratrol).	[190]
				[97]
				[94]
				[93]
				[96]
				[100]
				[95]
Alkaloids	•Anatabine, •Berberine, •CETA *(Corydalis edulis* total alkaloids), •Dauricine, •Deoxyvasicine, •Glaucocalyxin B, •Harmaline and Harmine.	•MKA (*Murraya koenigii* girinimbine, mahanimbine, murrayanine), • Neferine, •Oridonin, •Piperine, •Retinoic acid, •Tetrandrine, •Trigonelline.		[97]
				[94]
				[93]
Terpenes, Terpenoids	•Artemether, •α-cyperone, •Bakkenolide B, •Bilobalide, •Carnosic acid, •Dihydroasparagusic acid, •Geniposide, •Ginkgolide, •Ginsenosides,	•Lactucopicrin, •Linalool, •Paeoniflorin, •Tanshinone IIA, •Xanthoceraside, •7β-(3-ethylcis-crotonoyloxy)-1α-(2-methylbutyryloxy)3,14-dehydro-Z-notonipetranone (ECN), •l-Theranine, •1,8-Cineole.	Promising (Ginseng).	[97]
				[94]
				[93]
				[95]
Carotenoids (tetraterpenoids)	•Astaxanthin, •Crocin,	•Lycopene, •Strigolactone.	One trial (Astaxanthin).	[97]
				[191]
Sulfur-Containing Secondary Metabolites	•Sulforaphane,	•Thiacremonone.		[97]
				[94]
				[105]
Phytocannabinoids	•Cannabidiol.		One trial on BPSD.	[192]
				[95]
				[193]
				[194]
Miscellaneous Compounds	•Kavalactone (Methysticin), •Mushrooms (*Coriolus versicolor*, *Hericium erinaceus*), •Naphthoquinone (shikonin),	•Omega-3 (n-3) Essential Fatty Acids, •Polysaccharide (Chitosan), •Vitamins (α-Tocopherol, Quinine).	Promising (Docosahexaenoic acid, DHA, α-Lipoic Acid, ALA). Promising (α-Tocopherol, in combination).	[97]
				[195]
				[100]
				[196]
Gut Microbiota Regulators	•Probiotics, •sodium oligomannate (GV-971).		Promising (Probiotics). Promising (GV-971).	[190]
				[197]

BPSD = Behavioral And Psychological Symptoms In Dementia

## Curcumin

Among natural compounds, curcumin is one of the best known. Curcumin has been found to modulate the activity of several key transcription factors including NF-κB, Keap1/Nrf2, AP-1, androgen receptor (AR) and AR-related cofactors, and, in turn, the cellular expression profiles [99]. Curcumin also shows anti-depression (alters HPA axis) activity by lowering cortisol, inhibiting monoamine oxidase A and B and improves neurotrophic factors activity [100]. Anti-amyloidogenic property or anti-protein aggregation/misfolding (Aβ amyloid/β secretase, α-synuclein, τ, and prions protein) of curcumin was reported through modulating PPAR-γ (Peroxisome proliferator-activated receptor-γ), restoring innate immune system (*via* type I macrophages) as well as autophagy machinery and ubiquitin-proteasome system [100]. In a study, AD patients treated with curcumin showed increased β-amyloid protein clearance and improved phagocytosis of amyloid plaques [101].

Although several studies demonstrated these points, few clinical trials have been conducted, producing mixed results, and complicating their interpretation. These inconsistencies may be related to differences in methodology and in included population [102].

### Epigallocatechin-3-gallate (EGCG)

EGCG is one of the major catechins of green tea (polyphenol). EGCG enhances the neuronal growth factors (BDNF/GDNF) by inactivating microglial cells and improves antioxidant status *via* modulating Nrf2/HO-1 and NF-κB/JNK/MAPK signalling pathways [100]. The major neuroprotective function of EGCG is exerted by its anti-amyloidogenic property which includes the inhibition of Aβ1-42 amyloid fibril aggregation or production as well as suppresses alpha-secretase/Synuclein protein/peptide misfolding *via* modulating apoptosis and autophagy pathways [100]. ECGC can promote changes in steroid hormones release [103].

The most recent meta-analysis conducted by Kakutani and his colleagues [104] displayed, in wide samples positive neuroprotective effect of ECGC by reducing the risk of dementia, AD, and cognitive impairment [95].

## Ginkgo biloba

Leaf extracts of *Ginkgo biloba* are widely used for AD. The principal bioactive components of *ginkgo* include terpenoids *(e.g*. ginkgolide and bilobalide) and flavonol glycosides (e.g. quercetin and kaempferol). Its effects on Keap1/Nrf2 [105] and/or NF-κB [106] have been reported. Amyloidogenesis and Aβ aggregation, modulation of phosphorylation of τ protein, ion homeostasis, and even induction of growth factors are possible mechanisms of action of *Ginkgo biloba* [107].

In a randomised, double-blind, placebo-controlled trial, 216 participants with AD received a 24-weeks treatment of a standardised *ginkgo* extract, obtaining an improvement in attention and memory function [108]. In a more recent study, 24-weeks treatment with the *ginkgo* extract was associated with a significant improvement in cognitive functions and neuropsychiatric symptoms in 404 AD patients [109]. *Ginkgo*’s beneficial therapeutic effects have been demonstrated in several systematic reviews and meta-analyses that showed *ginkgo* treatment stabilised or slowed decline in cognition, function, and behaviour, neuropsychiatric symptoms in dementia patients [110-114]. A recent updated review of randomized clinical trials concluded that *Ginkgo biloba* may be able to improve the cognitive functions in patients who suffered from mild dementia by a long-term administration (more than 24 weeks) and by using an appropriate dosage (240 mg per day) [115].

## Resveratrol

Resveratrol is one of the popular nutraceuticals as it shows various health-promoting properties. Resveratrol notably improves BBB integrity *via* enhancing Nrf2/HO-1 antioxidant system and PI3K/Akt signalling pathway as well as effectively attenuates the inflammatory response *via* regulating NF-κB and JNK/MAPK system [100]. The major neuroprotective property of resveratrol is by acting as anti-protein aggregation/misfolding or anti-amyloidogenic property (anti-amyloidogenesis) by abolishing the neurofibrillary τ protein tangles or Aβ protein formation and deposition (hyperphosphorylation) and thereby improves cognition functions [100]. Resveratrol can enhance estrogen levels in an AD model [116].

Two clinical trials have been performed for resveratrol in AD [117, 118] demonstrating that it was safe and well-tolerated and can penetrate the BBB to have CNS effects.

## Polyunsaturated fatty acids (omega-3- fatty acids, ω-3 FA)

ω-3 FA are classified as essential fatty acids as they cannot be synthesized by a human. They display anti-inflammatory properties by inactivating microglia/astrocytes *via* JNK and PPAR-γ signalling pathway. Anti-inflammation activities have been demonstrated also *via* Keap1/Nrf2 and NF-κB systems [119]. ω-3 FA mitigate amyloid β plaque as well as aggregation of τ protein (hyperphosphorylation) *via* enhancing α-β 42 phagocytosis. Moreover, ω-3 FA inhibit β/γ secretase enzyme (anti-amyloidogenic), enhance neurotransmitter production and improve neurogenesis, producing neurotrophic growth factors [100].

Morris *et al*. [120] conducted a clinical trial by including 131 AD subjects and found that consumption of fish oil rich in Docosahexaenoic acid (DHA) could considerably lower (60%) the risk of incident AD. A meta-analysis also showed that higher consumption of fish oil was associated with a 36% reduction in the risk of AD. In another study, increment (by 100 g) in fish oil consumption was associate with further 11% reduction in the risk of AD [121]. Lee and others [122] conducted a randomized, double-blind, placebo-control trial by supplementation of DHA (fish oil) where they demonstrated that DHA considerably improved the working and verbal memory in MCI patients. Two open-label studies reported that α-Lipoic Acid (ALA) supplementation benefited patients with AD [123, 124]. More recently, it has been demonstrated that the effect of ω3-FA supplementation on cognitive functions appeared to be influenced by baseline total homocysteine, suggesting that adequate B vitamin status is required to obtain beneficial effects of ω3-FA on cognition [125].

## Ginseng

The root of *Panax ginseng* (ginseng) has been traditionally used for centuries to manage a wide range of disorders, including AD, in many Asian countries. Studies showed activities of ginseng by Keap1/Nrf2 [126] and NF-κB [127].

In three small, open-label trials, 12 and 24 weeks of ginseng treatment significantly improved Alzheimer’s Disease Assessment Scale-cognitive subscale (ADAS-cog) and Mini-Mental State Examination (MMSE) scores in patients with AD [128-130].

## Genistein

Genistein, an isoflavone mainly found in soy products, is a potentially effective compound in preventing AD. It can downregulate TNF-α, IL-1β, TLR4, NF-κB, promote the upregulation of IB-κ [131] and act on Keap1/Nrf2 system [132]. Genistein protects Aβ-induced neurotoxicity by inducing the PKC (protein kinase C) signalling pathway, which further regulates the activities of α- and β-secretases and thereby inhibits the formation and toxicity of Aβ [131]. Genistein has the structure and function like 17β-estradiol [133] and it can attach itself to the receptive proteins of the female sex hormones. Even being able to replace them, genistein can have either an estrogenic or a regulatory hormonal role [134].

One trial reported that six months of treatment with soy isoflavones did not benefit cognition in AD [135].

## Gut microbiota (GM) regulators

Several studies highlighted an intricate association between GM alterations and amyloid formation, increased systemic inflammation responses and cognitive impartment in AD, suggesting that modulation of GM with specific GM regulators could be a promising therapy to alleviate its underlying symptoms [136] (Fig. 2).

It has been over 17 years since the scientific definition of Probiotics (PBs) was introduced with guidelines to ensure appropriate use of the term. An expert panel defined PBs as “live microorganisms which when administered in adequate amounts confer a health benefit on the host”. In 2014, a consensus panel made a small change replacing “which” with “that”. Reid *et al*. 2016 [137] have recently urged researchers to follow the precise definition and to stay consistent in order to help advance the development and validation of microbial therapies [138]. PBs organisms are crucial for the maintenance of balance in human intestinal microbiota. Numerous scientific reports confirmed their positive effects in the host’s health. It has been demonstrated that PBs decreased hypothalamic expression levels of the pro-neuroinflammatory factors like IL-1β, NLRP3 inflammasome, Caspase-1 and NF-κB [139], demonstrating anti-inflammatory and pro-immune activities.

At present, few clinical trials have been conducted in an AD population. In a study [140], the authors demonstrated that PBs and selenium co-supplementation for 12 weeks to patients with AD improved cognitive functions and some metabolic profiles. In another study [141], the authors showed that oral supplementation of *Bifidobacterium breve A1 (B. breve A1)* in participants with MCI improved cognitive functions.

Sodium oligomannate (GV-971) is an orally administered mixture of acidic linear oligosaccharides derived from marine brown algae. GV-971 was developed by Shanghai Green Valley Pharmaceuticals for the treatment of AD and was approved by China’s regulators for the treatment of mild-to-moderate AD in November 2019.

A study has demonstrated solid and consistent cognition improvement in a phase 3 clinical trial in China, where GV-971 suppresses gut dysbiosis and the associated phenylalanine/isoleucine accumulation, harnesses neuroinflammation and reverses the cognition impairment. In addition, GV-971 can easily penetrate the BBB to directly bind to Aβ and inhibit Aβ fibril formation [142, 143].

Despite this wide recent literature on the potential effectiveness of nutraceuticals and AD, some limitations must be underlined. One of the major limits is linked to their low bioavailability and rapid metabolism to secondary metabolites (i.e crossing BBB) as well as lack of strong pharmacokinetics/dynamic data (dose, stability, toxicity, effective administration route) [100].

For instance, tea catechins [144, 145], resveratrol [146], and curcumin [147-150] have been reported to show low bioavailability and limited stability as these natural compounds are sensitive to the degradation or transformation to inactive derivates [151, 152]. Consequently, this can limit their effectiveness.

From this prospective, we present the potentiality of a promising therapy that similarly attenuates NF-κB and potentiates Keap1/Nrf2, represented by the O_2_-O_3_ therapy. This treatment is never tested till now in AD, but that in many other diseases where the inflammation processes are one of the main causes, it has demonstrated significant positive effects.

**Figure 4.**
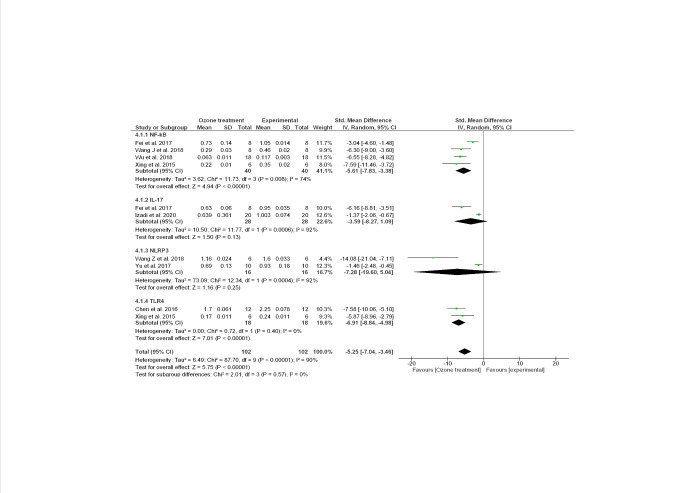
Forest plot for odds ratio (OR) from meta-analysis of the inflammation-NF-κB (nuclear factor kappa-light-chain-enhancer of activated B cells)/NLRP3 inflammasome/Toll-Like Receptor 4 (TLR4)/Interleukin IL-17α signalling (mRNA genes expression) before and after ozone (O_3_) treatment. CI, confidence interval; Chi2, χ2 test of goodness of fit; Tau2, estimate of the between-study variance in a random-effects meta-analysis.

## Oxygen-Ozone (O_2_-O_3_) therapy

O_3_ is a triatomic gaseous molecule which has been used as a powerful oxidant in medicine for more than 150 years [153]. Some important studies demonstrated that neutrophils could catalyse the generation of O_3_ by a water oxidation pathway, leading to an efficient killing of bacteria [154, 155].

The O_2_-O_3_ therapy is a non-invasive, non-pharmacological, no-side effect and low-cost procedure applied in medicine for the treatment of more than 50 pathological processes. O_3_ has a dose/effect relationship, and it is known for its antiseptic and therapeutic effects determined by the hormesis theory ([156-158] for review [27]). The mechanism of action of systemic O_3_ therapy is an “indirect” effect. O_3_ does not follow the standard principles of Pharmacology: absorption, distribution, metabolism and excretion. O_3_ “only acts” as a modulator or pro-drug and, by inducing secondary messengers, will enhance the subsequent adaptive responses. After this fast reaction (few seconds), O_3_ disappears.

O_3_ concentration and effects do not follow a linear relationship: very low concentrations could have no effect, whereas very high concentrations can lead to contrary effects to those produced by lower/middle concentrations [159]. It has been demonstrated that the therapeutic efficacy of O_2_-O_3_ may be partly due to the controlled and moderate oxidative stress produced by the reaction of O_3_ with several biological components [160]. At least 65 findings reviewed in Scassellati *et al*. [27] demonstrated the preconditioning/postconditioning of O_3_ on endogenous pro-antioxidant mechanisms *in vivo* on animal models and *in vitro* on cells. We observed that postconditioning of O_3_ exerts a protective effect on ischemia- reperfusion injury (IRI) in rat models of cochlear, hepatic, intestinal, renal, cardiac, lung and skeletal ischemia through an oxidative pre-conditioning mechanism that prevents the increase of the endogenous pro-oxidant and stimulates antioxidant mechanisms. O_3_ postconditioning prevents also other different kind of injury: LPS injection, carbon tetrachloride, partial hepatectomy, total body irradiation, methotrexate, intraperitoneal injection of rat fecal material, sepsis, kidney and cardiac transplantation, contrast-induced nephropathy, induction of diabetes, cisplatin-induced nephrotoxicity, contrast-induced nephropathy agent, hydrogen peroxide, H_2_O_2_, doxorubicin, ototoxicity, noise exposure, hypothermia, lipofundin [27].

## Biological activities of the ozone (O_3_)

O_3_, when dissolved in plasma, reacts with a series of biomolecules and then disappears. There are two compounds: ROS and lipid oxidation products or LOPs, that represent the “ozone messengers”. They are responsible for its biological and therapeutic effects. ROS are produced immediately in the initial phase (mainly H_2_O_2_) and are responsible for the early biological effects on the blood (erythrocytes, leukocytes, platelets). In contrast, LOPs (i.e. 4-hydroxynonenal, 4HNE), which are produced simultaneously, have a longer half-life, reach the vascular system, and interact with various organs, where they trigger late effects [161-165] (Fig. 3).

## Anti-inflammatory features

**Figure 5.**
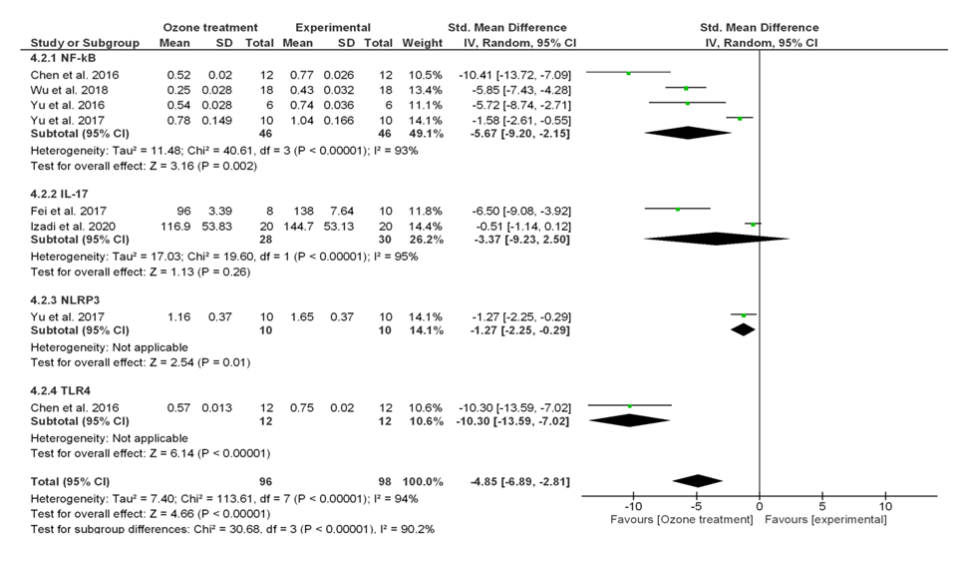
Forest plot for odds ratio (OR) from meta-analysis of the inflammation-NF-κB (nuclear factor kappa-light-chain-enhancer of activated B cells)/NLRP3 inflammasome/Toll-Like Receptor 4 (TLR4)/Interleukin IL-17α signalling (protein levels) before and after ozone (O_3_) treatment. CI, confidence interval; Chi2, χ2 test of goodness of fit; Tau2, estimate of the between-study variance in a random-effects meta-analysis.

The presence of H_2_O_2_ and 4HNE determined by mild ozonization [166-169], causes the blocking of the action of NF-κB, determining a reduction of the inflammation and apoptotic cell death. In this way, O_3_ can exert its anti-inflammatory ability (Fig. 3). As reported, NF-κB along with Keap1/Nrf2 are the major signalling pathways modulating inflammation, and their cross-talk brings to coordinated inflammatory responses [170]. In our previous work [27], we demonstrated by meta-analytic approaches, the significant effects of O_3_ on Keap1/Nrf2 system, evidencing an increased expression levels of this molecule after O_3_ administration. Here, we wanted to demonstrate the modulatory effect of O_3_ on NF-κB/NLRP3 inflammasome/TLR4/IL-17α inflammatory processes. By meta-analytic tools (supplementary Table 2), we found that O_3_ decreased NF-κB/NLRP3 inflammasome/TLR4/IL-17α mRNA expression (Fig. 4 Random model Z=5.75 p<0.00001 Odd Ratio (OR)=-5.25 95%CI:-7.04/-3.46 even after Bonferroni correction 0.05/4 = 0.01) and protein (Fig. 5, Random model Z=4.66 p<0.00001 OR=-4.85 95%CI:-6.89/-2.81 even after Bonferroni correction) levels. Considering single markers with major studies available, we found reduced levels of NF-κB both at mRNA expression (Fig. 4, Z=4.94 p<0.00001 OR=-5.61 95%CI:-7.83/-3.38) and protein (Fig. 5, Z=3.16 p=0.002 OR=-5.67 95%CI:-9.20/-2.15) levels after O_3_ treatment. High heterogeneity in effect size across the studies (Fig. 4, mRNA expression p<0.00001 I^2^=90%; Fig. 5, protein levels p<0.00001 I^2^=94%) was observed in these meta-analyses. This is essentially explained by the presence of factors such as the type of injury performed, different concentrations of O_3_ and duration time of treatments that influence the analyses (supplementary Table 2).

Fernández-Cuadros observed that O_3_ is able of modulating inflammation, decreasing inflammation markers such as CRP (C-reactive protein) and ESR (erythrocyte sedimentation rate) [171].

## Immunoregulation properties

H_2_O_2_ and 4HNE, after mild ozonization, can activate the transcription factors NFAT (nuclear factor activated T-cells) and/or AP-1, and in this way O_3_ stimulates the innate immune system, helping the cells to survive injury. IL-2, IL-6, IL-8, TNF-α, and IFN-γ produced by this mechanism will recruit neutrophils, lymphocytes, and macrophages, in order to carry out phagocytosis to limit infection at that level, killing local pathogens [153, 172-175]. Low doses of O_3_ have been shown to increase secretions of macrophages and leukocytes [176], to enhance the phagocytic capacity of granulocytes and to facilitate the formation of monocytes and activation of T-cells.

The mechanisms by which NFAT and AP-1 exercise their functions on immune responses are the following. A tyrosine-phosphorylation response takes place immediately in the ZAP-70 molecule when the T-cell antigen receptor (TCR) recognizes any invaders, and then activates phospholipase Cγ1 (PLCγ1) [177]. Membrane lipid phosphatidylinositol-4,5-bisphosphate (PIP2) can be hydrolysed by the activation of PLCγ1, therefore, producing two critical second messengers: inositol triphosphate (IP3) and diacylglycerol (DAG). Then, IP3 binds to its receptor (IP3r) located in the endoplasmic reticulum (ER) membrane, leading to Ca^2+^ from ER into the cytosol. The elevated levels of Ca^2+^ in cytosol will activate calcineurin, which dephosphorylates NFAT and transports it into the nucleus. NFAT then induces the transcription of cytokines implicated in the immune response [178].

PLCγ1 can also activate Ras/Mitogen-activated Protein Kinases (MAPK) signalling to promote the translocation of the AP-1 in the nucleus and activate the immune response [179].

## Effects on Glucocorticoids (GCs)

It has been demonstrated that administrated O_3_ also induces a release of DHEA, adrenocorticotropic hormone (ACTH), cortisol and corticotropin-releasing hormone (CRH), and endorphins [180].

## Anti-microbial properties

As summarized in [26], different findings support its anti-microbial activity. In bacteria, O_3_ disrupts the integrity of the bacterial cell wall through oxidation of phospholipids and lipoproteins [181, 182]. In fungi, O_3_ is able to inhibit the fungi’s growth, interacting in the same way as bacteria [183, 184]. In viruses, O_3_ damages the viral capsid and breaks the reproductive cycle by disrupting the contact between the virus and the cell through the process of peroxidation. It has been reported the efficacy of O_3_ also against the new pandemic COVID-19 [185, 186]. Figure 2 shows the potential mechanism of action of O_3_ at the GM level, suggesting that O_3_ can be an additional potential molecule acting as GM regulator, in synergy with PBs and GV-971.

## Future Directions and Conclusions

In this review, we support the wide literature on the crucial role that different nutraceuticals can play in neuroinflammation/steroid hormones alteration/neurodegeneration, directly or indirectly through the manipulation of GM, although further and future clinical studies are needed in AD. In addition, we present extensively research and strengths on immune/anti-inflammation/anti-microbial and pro-neuroendocrine activities of O_3_ that could have a significant clinical impact on AD, alone or in combination with the described nutraceuticals. With a view to prevention, these therapies, utilized in early stage could modulate those physiological mechanisms linked to aging and to cognitive and neuropsychiatric decline, that are potential risk factors to develop more severe neurodegeneration damage.

Challenges regarding treatments efficacy and costs still persist for AD. Correlated with the well-known interactions with NF-κB, Keap1/Nrf2 but also NFAT or AP-1 [166-168, 172] or steroid hormones system, GCs [174, 180], O_3_ and/or in combination with the other nutraceuticals described in Table 1 can potentially increase the immune system, decrease the neuroinflammation and modulate the neuroendocrine system, to consequently improve/resolve the cognitive performance and neuropsychiatric symptoms. These approaches could represent useful, safe, no-invasive, no-pharmacological, economical, effective treatments for these neurodegenerative conditions. The mechanisms of the positive effects of all nutraceuticals including O_3_ are attributed not only to up-regulation of cellular immune and anti-inflammation properties, but also to other crucial roles linked to anti-oxidant, anti-apoptosis, pro-autophagy, regeneration processes. In the specific case of O_3_, studies demonstrated enhancement in the release of growth factors from platelets, along with being a potent bactericide, fungicide and virucidal with potential effect on GM [27]. Moreover, ozonized erythrocytes show improved glycolysis with increased levels of ATP and 2,3-DPG (diphosphoglycerate), which can shift the HbO_2_ dissociation curve to the right, increase arterial PO_2_, and decrease venous PO_2_ (Bhor effect), improving oxygen supply to ischemic tissues [187, 188]. Continuous applications of O_3_ stimulate the bone marrow and induce it to generate new “gifted erythrocytes” with an increase in the content of 2,3-DPG, as well as an elevation of glucose 6-phosphate dehydrogenase (G6PD); this may allow a profound modification of functional activities leading tissues and organs from a hypoxic to a normoxic state [187, 188]. In the medical setting, this therapy employs a gas mixture of O_2_/O_3_, obtained from the modification of medical-grade O_2_ using certificated O_3_ generator device [188]. Based on the basic mechanisms of action of O_3_ in blood, the therapeutic range of O_3_ has been precisely calculated and found to be 10-80 μg/ml of O_3_ in blood [189]. Schwart-Tapia *et al*. [189] described widely different and main routes of application with relative concentrations of O_3_. O_3_ medical preparations are classified into three types: ozonized water, ozonized oil and ozonized gas. The side effects are minimal; the World Federation of Ozone therapy (WFOT) estimates the incidence of complications at 0.0007%. Moreover, the treatment is not only perfectly tolerated but most of patients have reported a feeling of wellness and euphoria throughout the cycle. This fact explains why the compliance of the patients remains excellent throughout the years.

As further studies are needed, we suggest that a strategic intervention treatment where the administration of different nutraceuticals and GM regulators potentiated with O_3_ at mild concentrations could represent convenient, inexpensive therapies, working in absence of side effects that will permit to modulate the immune, inflammatory, neuroendocrine and GM composition, impaired in AD. This will contribute to the paradigm shift from a single-target to a multi-target drug approach, especially for chronic and complex ailments. At the same time, metatranscriptomic, metabolomic and proteomic technologies are high throughput approaches needed to assess the effects of O_2_-O_3_ therapy and nutraceuticals intervention showing that a deeper integration of "omics" sciences along with more accurate clinical profiles and new computational methods will allow us to identify biomarkers associated to these therapies.

## Supplementary Materials

The Supplemenantry data can be found online at: www.aginganddisease.org/EN/10.14336/AD.2021.0122.


