# Supplementary material for: Promising Intervention Approaches to Potentially Resolve Neuroinflammation And Steroid Hormones Alterations in Alzheimer’s Disease and Its Neuropsychiatric Symptoms
Source: Aging Dis. 2021 Aug 1;12(5):1337–57. doi: 10.14336/AD.2021.0122 (PMC8279527; doi:10.14336/AD.2021.0122)
Supplement: Supplementary file 1 [file AD-12-5-1337-s.pdf]

Promising Intervention Approaches to Potentially Resolve Neuroinflammation And Steroid Hormones Alterations in Alzheimer's Disease and Its Neuropsychiatric Symptoms

Catia Scassellati[1*], Antonio Carlo Galoforo[2,3], Ciro Esposito[4,5,6], Miriam Ciani[7], Giovanni Ricevuti[6,8,9,#], Cristian Bonvicini[7,#]

© 2021. Scassellati C et al. Published online at http://www.aginganddisease.org/EN/10.14336/AD.2021.0122

SUPPLEMENTARY DATA

MATERIAL AND METHODS

1. Search strategy

We searched the electronic databases PubMed and Google scholar databases. The research was conducted with no restrictions on language. In PubMed, we used the search terms “natural compounds” OR “nutraceuticals” OR “phytochemicals” AND “Alzheimer’s Disease” OR “Alzheimer” OR “Alzheimer’s”. We selected articles that met the following inclusion criteria: (a) only reviews; (b) each study performed on human, animal models, *in vitro* approaches.

Starting from 1,832 records without time restriction, this research produced over 119 reviews on the topic in the last two years 2019-2020 (Table 1S).

2. Search strategy and selection criteria for meta-analyses

We searched the electronic databases PubMed, Google scholar, Cochrane Library databases. The research was conducted up to December 2020, with no restrictions on language, date, or study setting. In PubMed, we used the search terms “ozone therapy” OR “oxygen ozone therapy” AND “NF-κB” OR “inflammation” OR “anti-inflammatory” OR “cytokines” OR “interleukins” OR “inflammasome” OR “NLPR3” OR “NLR family pyrin domain containing 3” OR “TLR4” OR “Toll-like receptor 4” OR “Interleukin 17α” OR “IL 17α”.

Only the keyword “ozone therapy” in Pubmed produces 3,770 articles.

Inclusion and exclusion criteria

We selected articles that met the following inclusion criteria: (a) each type of pathology; (b) each study performed on animal models studies; (c) *in vitro* studies on cells; (d) studies on human; (e) each study performed on peripheral levels (protein); (f) each study performed on expression mRNA levels. The meta-analytic analyses were conducted where data were available before and after the O_3 treatments. We excluded: (a) studies regarding ozone as air pollutant or ozone-induced toxicity; (b) genetic studies on ozone exposition; (c) reviews, commentaries on the topic.

Data extraction for meta-analyses

CS and CB independently extracted the following data: authors of the studies, biomarkers, model, sample size (N), experiments, treatments, and dosages (Table 2S).

Statistical analyses

Review Manager was used to perform the meta-analysis (RevMan Version 5.1.6; Copenhagen, The Nordic Cochrane Centre, The Cochrane Collaboration, 2008). We used the random-effects model to generate a pooled effect size and 95% confidence interval (CI) from individual study effect sizes (the standardized mean difference using the inverse variance methods). The significance of the pooled effect sizes was determined by z-tests. Between-study heterogeneity was assessed using a χ^2 test of goodness of fit test and the I^2 statistic. We used a P-value < 0.05 to indicate statistical significance.

For multiple corrections, we applied the Bonferroni correction: 0.05/number of the biomarkers analysed.

Supplementary Table 1. List of 119 reviews on the “Natural Compounds” and “Alzheimer’s Disease” in the last two years 2019-2020.

A. 2020 year

1. Ahmad S, Campos MG, Fratini F, Altaye SZ, Li J. New Insights into the Biological and Pharmaceutical Properties of Royal Jelly. Int J Mol Sci. 2020 Jan 8;21(2):382. doi: 10.3390/ijms21020382. PMID: 31936187; PMCID: PMC7014095.
2. Alausa A, Ogundepo S, Olaleke B, Adeyemi R, Olatinwo M, Ismail A. Chinese nutraceuticals and physical activity; their role in neurodegenerative tauopathies. Chin Med. 2021 Jan 6;16(1):1. doi: 10.1186/s13020-020-00418-7. PMID: 33407732; PMCID: PMC7789572.
3. Ali AM, Kunugi H. Royal Jelly as an Intelligent Anti-Aging Agent-A Focus on Cognitive Aging and Alzheimer's Disease: A Review. Antioxidants (Basel). 2020 Sep 29;9(10):937. doi: 10.3390/antiox9100937. PMID: 33003559; PMCID: PMC7601550.
4. Ansari F, Pourjafar H, Tabrizi A, Homayouni A. The Effects of Probiotics and Prebiotics on Mental Disorders: A Review on Depression, Anxiety, Alzheimer, and Autism Spectrum Disorders. Curr Pharm Biotechnol. 2020;21(7):555-565. doi: 10.2174/1389201021666200107113812. PMID: 31914909.

5. Atlante A, Amadoro G, Bobba A, Latina V. Functional Foods: An Approach to Modulate Molecular Mechanisms of Alzheimer's Disease. Cells. 2020 Oct 23;9(11):2347. doi: 10.3390/cells9112347. PMID: 33114170; PMCID: PMC7690784.
6. Batiha GE, Beshbishy AM, Ikram M, Mulla ZS, El-Hack MEA, Taha AE, Algammal AM, Elewa YHA. The Pharmacological Activity, Biochemical Properties, and Pharmacokinetics of the Major Natural Polyphenolic Flavonoid: Quercetin. Foods. 2020 Mar 23;9(3):374. doi: 10.3390/foods9030374.
7. Bhatt T, Patel K. Carotenoids: Potent to Prevent Diseases Review. Nat Prod Bioprospect 2020 Jun;10(3):109-117. doi: 10.1007/s13659-020-00244-2.
8. Bhatti GK, Reddy AP, Reddy PH, Bhatti JS. Lifestyle Modifications and Nutritional Interventions in Aging-Associated Cognitive Decline and Alzheimer's Disease. Front Aging Neurosci. 2020 Jan 10;11:369. doi: 10.3389/fnagi.2019.00369. PMID: 31998117; PMCID: PMC6966236.
9. Breijyeh Z, Karaman R. Comprehensive Review on Alzheimer's Disease: Causes and Treatment. Molecules. 2020 Dec 8;25(24):5789.
10. Brimson JM, Prasanth MI, Malar DS, Brimson S, Tencomnao T. Rhinacanthus nasutus "Tea" Infusions and the Medicinal Benefits of the Constituent Phytochemicals. Nutrients. 2020 Dec 9;12(12):3776. doi: 10.3390/nu12123776.
11. Calabrese EJ, Kozumbo WJ. The phytoprotective agent sulforaphane prevents inflammatory degenerative diseases and age-related pathologies via Nrf2-mediated hormesis. Pharmacol Res. 2020 Nov 4:105283. doi: 10.1016/j.phrs.2020.105283. Epub ahead of print. PMID: 33160067.
12. Calabrese EJ. Hormesis and Ginseng: Ginseng Mixtures and Individual Constituents Commonly Display Hormesis Dose Responses, Especially for Neuroprotective Effects. Molecules. 2020 Jun 11;25(11):2719. doi: 10.3390/molecules25112719. PMID: 32545419; PMCID: PMC7321326.
13. Calfio C, Gonzalez A, Singh SK, Rojo LE, Maccioni RB. The Emerging Role of Nutraceuticals and Phytochemicals in the Prevention and Treatment of Alzheimer's Disease. J Alzheimers Dis. 2020;77(1):33-51. doi: 10.3233/JAD-200443. PMID: 32651325.
14. Cassidy L, Fernandez F, Johnson JB, Naiker M, Owoola AG, Broszczak DA. Oxidative stress in alzheimer's disease: A review on emergent natural polyphenolic therapeutics. Complement Ther Med. 2020 Mar;49:102294. doi: 10.1016/j.ctim.2019.102294.
15. Chatterjee P, Fernando M, Fernando B, Dias CB, Shah T, Silva R, Williams S, Pedrini S, Hillebrandt H, Goozee K, Barin E, Sohrabi HR, Garg M, Cunnane S, Martins RN. Potential of coconut oil and medium chain triglycerides in the prevention and treatment of Alzheimer's disease. Mech Ageing Dev. 2020 Mar;186:111209. doi: 10.1016/j.mad.2020.111209. Epub 2020 Jan 15. PMID: 31953123.
16. Chauhan A, Chauhan V. Beneficial Effects of Walnuts on Cognition and Brain Health. Nutrients. 2020 Feb 20;12(2):550. doi: 10.3390/nu12020550. PMID: 32093220; PMCID: PMC7071526.
17. Chen DB, Gao HW, Peng C, Pei SQ, Dai AR, Yu XT, Zhou P, Wang Y, Cai B. Quinones as preventive agents in Alzheimer's diseases: focus on NLRP3 inflammasomes. J Pharm Pharmacol. 2020 Nov;72(11):1481-1490. doi: 10.1111/jphp.13332.
18. Chiu HF, Venkatakrishnan K, Wang CK. The role of nutraceuticals as a complementary therapy against various neurodegenerative diseases: A mini-review. J Tradit Complement Med. 2020 Apr 1;10(5):434-439. doi: 10.1016/j.jtcme.2020.03.008. PMID: 32953558; PMCID: PMC7484964.
19. de Araújo FF, de Paulo Farias D, Neri-Numa IA, Pastore GM. Polyphenols and their applications: An approach in food chemistry and innovation potential. Food Chem. 2021 Feb 15;338:127535. doi: 10.1016/j.foodchem.2020.127535.
20. Devi SA, Chamoli A. Polyphenols as an Effective Therapeutic Intervention Against Cognitive Decline During Normal and Pathological Brain Aging. Adv Exp Med Biol. 2020;1260:159-174. doi: 10.1007/978-3-030-42667-5_7. PMID: 32304034.
21. Freyssin A, Page G, Fauconneau B, Rioux Bilan A. Natural stilbenes effects in animal models of Alzheimer's disease. Neural Regen Res. 2020 May;15(5):843-849. doi: 10.4103/1673-5374.268970.
22. Grodzicki W, Dziendzikowska K. The Role of Selected Bioactive Compounds in the Prevention of Alzheimer's Disease. Antioxidants (Basel). 2020 Mar 11;9(3):229. doi: 10.3390/antiox9030229.
23. Gruendler R, Hippe B, Sendula Jengic V, Peterlin B, Haslberger AG. Nutraceutical Approaches of Autophagy and Neuroinflammation in Alzheimer's Disease: A Systematic Review. Molecules. 2020 Dec 18;25(24):6018. doi: 10.3390/molecules25246018. PMID: 33353228; PMCID: PMC7765980.
24. Gupta J, Gupta R. Nutraceutical Status and Scientific Strategies for Enhancing Production of Omega-3 Fatty Acids from Microalgae and their Role in Healthcare. Curr Pharm Biotechnol. 2020;21(15):1616-1631. doi: 10.2174/1389201021666200703201014. PMID: 32619166.
25. Hanafy DM, Burrows GE, Prenzler PD, Hill RA. Potential Role of Phenolic Extracts of Mentha in Managing Oxidative Stress and Alzheimer's Disease. Antioxidants (Basel). 2020 Jul 17;9(7):631. doi: 10.3390/antiox9070631.

© 2021. Scassellati C et al. Published online at http://www.aginganddisease.org/EN/10.14336/AD.2021.0122

26. Howes MR, Perry NSL, Vásquez-Londoño C, Perry EK. Role of phytochemicals as nutraceuticals for cognitive functions affected in ageing. Br J Pharmacol. 2020 Mar;177(6):1294-1315. doi: 10.1111/bph.14898. Epub 2020 Feb 3. PMID: 31650528; PMCID: PMC7056459.
27. Hung WL, Ho CT, Pan MH. Targeting the NLRP3 Inflammasome in Neuroinflammation: Health Promoting Effects of Dietary Phytochemicals in Neurological Disorders. Mol Nutr Food Res. 2020 Feb;64(4):e1900550. doi: 10.1002/mnfr.201900550.
28. Irfan M, Kwak YS, Han CK, Hyun SH, Rhee MH. Adaptogenic effects of Panax ginseng on modulation of cardiovascular functions. J Ginseng Res. 2020 Jul;44(4):538-543. doi: 10.1016/j.jgr.2020.03.001. Epub 2020 Mar 28. PMID: 32617033; PMCID: PMC7322748.
29. Karaman Mayack B, Sippl W, Ntie-Kang F. Natural Products as Modulators of Sirtuins. Molecules. 2020 Jul 20;25(14):3287. doi: 10.3390/molecules25143287.
30. Kesika P, Suganthy N, Sivamaruthi BS, Chaiyasut C. Role of gut-brain axis, gut microbial composition, and probiotic intervention in Alzheimer's disease. Life Sci. 2021 Jan 1;264:118627. doi: 10.1016/j.lfs.2020.118627. Epub 2020 Oct 22. PMID: 33169684.
31. Khan A, Jahan S, Imtiyaz Z, Alshahrani S, Antar Makeen H, Mohammed Alshehri B, Kumar A, Arafah A, Rehman MU. Neuroprotection: Targeting Multiple Pathways by Naturally Occurring Phytochemicals. Biomedicines. 2020 Aug 12;8(8):284. doi: 10.3390/biomedicines8080284. PMID: 32806490; PMCID: PMC7459826.
32. Khan MS, Ikram M, Park JS, Park TJ, Kim MO. Gut Microbiota, Its Role in Induction of Alzheimer's Disease Pathology, and Possible Therapeutic Interventions: Special Focus on Anthocyanins. Cells. 2020 Apr 1;9(4):853. doi: 10.3390/cells9040853.
33. Kiani AK, Miggiano GAD, Aquilanti B, Velluti V, Matera G, Gagliardi L, Bertelli M. Food supplements based on palmitoylethanolamide plus hydroxytyrosol from olive tree or Bacopa monnieri extracts for neurological diseases. Acta Biomed. 2020 Nov 9;91(13-S):e2020007. doi: 10.23750/abm.v91i13-S.10582.
34. Kobayashi H, Murata M, Kawanishi S, Oikawa S. Polyphenols with Anti-Amyloid β Aggregation Show Potential Risk of Toxicity Via Pro-Oxidant Properties. Int J Mol Sci. 2020 May 18;21(10):3561. doi: 10.3390/ijms21103561.
35. Lakey-Beitia J, Burillo AM, La Penna G, Hegde ML, Rao KS. Polyphenols as Potential Metal Chelation Compounds Against Alzheimer's Disease. J Alzheimers Dis. 2020 Jun 15. doi: 10.3233/JAD-200185.
36. Leri M, Scuto M, Ontario ML, Calabrese V, Calabrese EJ, Bucciantini M, Stefani M. Healthy Effects of Plant Polyphenols: Molecular Mechanisms. Int J Mol Sci. 2020 Feb 13;21(4):1250. doi: 10.3390/ijms21041250. PMID: 32070025; PMCID: PMC7072974.
37. Liu S, Li X. Regulation of Autophagy in Neurodegenerative Diseases by Natural Products. Adv Exp Med Biol. 2020;1207:725-730. doi: 10.1007/978-981-15-4272-5_54.
38. López-Gambero AJ, Sanjuan C, Serrano-Castro PJ, Suárez J, Rodríguez de Fonseca F. The Biomedical Uses of Inositols: A Nutraceutical Approach to Metabolic Dysfunction in Aging and Neurodegenerative Diseases. Biomedicines. 2020 Aug 20;8(9):295. doi: 10.3390/biomedicines8090295. PMID: 32825356; PMCID: PMC7554709.
39. Maher PA. Using Plants as a Source of Potential Therapeutics for the Treatment of Alzheimer's Disease Yale J Biol Med 2020 Jun 29;93(2):365-373.
40. Manandhar S, Kabekkodu SP, Pai KSR. Aberrant canonical Wnt signaling: Phytochemical based modulation. Phytomedicine. 2020 May 23;76:153243. doi: 10.1016/j.phymed.2020.153243.
41. Maretzke F, Bechthold A, Egert S, Ernst JB, Melo van Lent D, Pilz S, Reichrath J, Stangl GI, Stehle P, Volkert D, Wagner M, Waizenegger J, Zittermann A, Linseisen J. Role of Vitamin D in Preventing and Treating Selected Extraskeletal Diseases-An Umbrella Review. Nutrients. 2020 Mar 31;12(4):969. doi: 10.3390/nu12040969. PMID: 32244496; PMCID: PMC7231149.
42. Margină D, Ungurianu A, Purdel C, Nițulescu GM, Tsoukalas D, Sarandi E, Thanasoula M, Burykina TI, Tekos F, Buha A, Nikitovic D, Kouretas D, Tsatsakis AM. Analysis of the intricate effects of polyunsaturated fatty acids and polyphenols on inflammatory pathways in health and disease. Food Chem Toxicol. 2020 Sep;143:111558. doi: 10.1016/j.fct.2020.111558. Epub 2020 Jul 5. PMID: 32640331; PMCID: PMC7335494.
43. Martins M, Silva R, M M Pinto M, Sousa E. Marine Natural Products, Multitarget Therapy and Repurposed Agents in Alzheimer's Disease. Pharmaceuticals (Basel). 2020 Sep 11;13(9):242. doi: 10.3390/ph13090242.
44. Mateos R, Pérez-Correa JR, Domínguez H. Bioactive Properties of Marine Phenolics. Mar Drugs. 2020 Sep 30;18(10):501. doi: 10.3390/md18100501. PMID:33007997; PMCID: PMC7601137.
45. Meng X, Zhou J, Zhao CN, Gan RY, Li HB. Health Benefits and Molecular Mechanisms of Resveratrol: A Narrative Review. Foods. 2020 Mar 14;9(3):340. doi: 10.3390/foods9030340. PMID: 32183376; PMCID: PMC7143620.
46. Mohd Sairazi NS, Sirajudeen KNS. Natural Products and Their Bioactive Compounds: Neuroprotective Potentials against Neurodegenerative Diseases. Evid Based Complement Alternat Med. 2020 Feb 14;2020:6565396. doi: 10.1155/2020/6565396.

47. Nourbakhsh F, Read MI, Barreto GE, Sahebkar A. Boosting the autophagy-lysosomal pathway by phytochemicals: A potential therapeutic strategy against Alzheimer's disease. IUBMB Life. 2020 Nov;72(11):2360-2281. doi: 10.1002/iub.2369.
48. Novik G, Savich V. Beneficial microbiota. Probiotics and pharmaceutical products in functional nutrition and medicine. Microbes Infect. 2020 Jan-Feb;22(1):8-18. doi: 10.1016/j.micinf.2019.06.004. Epub 2019 Jun 21. PMID: 31233819.
49. Oppedisano F, Maiuolo J, Gliozzi M, Musolino V, Carresi C, Nucera S, Scicchitano M, Scarano F, Bosco F, Macrì R, Ruga S, Zito MC, Palma E, Muscoli C, Mollace V. The Potential for Natural Antioxidant Supplementation in the Early Stages of Neurodegenerative Disorders. Int J Mol Sci. 2020 Apr 9;21(7):2618. doi: 10.3390/ijms21072618. PMID: 32283806; PMCID: PMC7177481.
50. Patnode CD, Perdue LA, Rossom RC, Rushkin MC, Redmond N, Thomas RG, Lin JS. Screening for Cognitive Impairment in Older Adults: An Evidence Update for the U.S. Preventive Services Task Force [Internet]. Rockville (MD): Agency for Healthcare Research and Quality (US); 2020 Feb. Report No.: 19-05257-EF-1. PMID: 32129963.
51. Pogačnik L, Ota A, Ulrih NP. An Overview of Crucial Dietary Substances and Their Modes of Action for Prevention of Neurodegenerative Diseases. Cells. 2020 Feb 28;9(3):576. doi: 10.3390/cells9030576.
52. Qian J, Hou M, Wu X, Dai C, Sun J, Dong L. A review on the extraction, purification, detection, and pharmacological effects of 2,3,5,4'-tetrahydroxystilbene-2-O-β-d-glucoside from Polygonum multiflorum. Biomed Pharmacother. 2020 Apr;124:109923. doi: 10.1016/j.biopha.2020.109923.
53. Rahman MA, Rahman MR, Zaman T, Uddin MS, Islam R, Abdel-Daim MM, Rhim H. Emerging Potential of Naturally Occurring Autophagy Modulators Against Neurodegeneration. Curr Pharm Des. 2020;26(7):772-779. doi: 10.2174/1381612826666200107142541.
54. Ramalho MJ, Andrade S, Loureiro JA, do Carmo Pereira M. Nanotechnology to improve the Alzheimer's disease therapy with natural compounds. Drug Deliv Transl Res. 2020 Apr;10(2):380-402. doi: 10.1007/s13346-019-00694-3.
55. Ramli NZ, Yahaya MF, Tooyama I, Damanhuri HA. A Mechanistic Evaluation of Antioxidant Nutraceuticals on Their Potential against Age-Associated Neurodegenerative Diseases. Antioxidants (Basel). 2020 Oct 20;9(10):1019. doi: 10.3390/antiox9101019. PMID: 33092139; PMCID: PMC7588884.
56. Rebas E, Rzajew J, Radzik T, Zylinska L. Neuroprotective Polyphenols: A Modulatory Action on Neurotransmitter Pathways. Curr Neuropharmacol. 2020;18(5):431-445. doi: 10.2174/1570159X18666200106155127.
57. Ruthirakuhan M, Herrmann N, Andreazza AC, Verhoeff NPLG, Gallagher D, Black SE, Kiss A, Lanctôt KL. Agitation, Oxidative Stress, and Cytokines in Alzheimer Disease: Biomarker Analyses From a Clinical Trial With Nabilone for Agitation. J Geriatr Psychiatry Neurol. 2020 Jul;33(4):175-184. doi: 10.1177/0891988719874118.
58. Sánchez-Sarasúa S, Fernández-Pérez I, Espinosa-Fernández V, Sánchez-Pérez AM, Ledesma JC. Can We Treat Neuroinflammation in Alzheimer's Disease? Int J Mol Sci. 2020 Nov 19;21(22):8751. doi: 10.3390/ijms21228751. PMID: 33228179; PMCID: PMC7699542.
59. Serra D, Almeida LM, Dinis TCP. Polyphenols in the management of brain disorders: Modulation of the microbiota-gut-brain axis. Adv Food Nutr Res. 2020;91:1-27. doi: 10.1016/bs.afnr.2019.08.001. Epub 2019 Aug 14. PMID: 32035595.
60. Shakeri F, Bianconi V, Pirro M, Sahebkar A. Effects of Plant and Animal Natural Products on Mitophagy. Oxid Med Cell Longev. 2020 Mar 10;2020:6969402. doi: 10.1155/2020/6969402.
61. Sharifi-Rad M, Lankatillake C, Dias DA, Docea AO, Mahomoodally MF, Lobine D, Chazot PL, Kurt B, Tumer TB, Moreira AC, Sharopov F, Martorell M, Martins N, Cho WC, Calina D, Sharifi-Rad J. Impact of Natural Compounds on Neurodegenerative Disorders: From Preclinical to Pharmacotherapeutics. J Clin Med. 2020 Apr 8;9(4):1061. doi: 10.3390/jcm9041061.
62. Sharma S, Cooper R, Bhardwaj G, Cannom DS. The genus Nepeta: Traditional uses, phytochemicals and pharmacological properties. J Ethnopharmacol 2020 Dec 8;268:113679. doi: 10.1016/j.jep.2020.113679.
63. Stacchiotti A, Corsetti G. Natural Compounds and Autophagy: Allies Against Neurodegeneration. Front Cell Dev Biol. 2020 Sep 22;8:555409.
64. Sung PS, Lin PY, Liu CH, Su HC, Tsai KJ. Neuroinflammation and Neurogenesis in Alzheimer's Disease and Potential Therapeutic Approaches. Int J Mol Sci. 2020 Jan 21;21(3):701. doi: 10.3390/ijms21030701.
65. Tadokoro K, Ohta Y, Inufusa H, Loon AFN, Abe K. Prevention of Cognitive Decline in Alzheimer's Disease by Novel Antioxidative Supplements. Int J Mol Sci. 2020 Mar 13;21(6):1974. doi: 10.3390/ijms21061974. PMID: 32183152; PMCID: PMC7139972.
66. Uddin MS, Al Mamun A, Kabir MT, Ahmad J, Jeandet P, Sarwar MS, Ashraf GM, Aleya L. Neuroprotective role of polyphenols against oxidative stress-mediated neurodegeneration. Eur J Pharmacol. 2020 Nov 5;886:173412. doi: 10.1016/j.ejphar.2020.173412.
67. Uddin MS, Kabir MT, Niaz K, Jeandet P, Clément C, Mathew B, Rauf A, Rengasamy KRR, Sobarzo-Sánchez E, Ashraf GM, Aleya L. Molecular Insight into the Therapeutic Promise of Flavonoids against Alzheimer's Disease. Molecules. 2020 Mar 11;25(6):1267. doi: 10.3390/molecules25061267.

© 2021. Scassellati C et al. Published online at http://www.aginganddisease.org/EN/10.14336/AD.2021.0122

68. Waheed Janabi AH, Kamboh AA, Saeed M, Xiaoyu L, BiBi J, Majeed F, Naveed M, Mughal MJ, Korejo NA, Kamboh R, Alagawany M, Lv H. Flavonoid-rich foods (FRF): A promising nutraceutical approach against lifespan-shortening diseases. Iran J Basic Med Sci. 2020 Feb;23(2):140-153. doi: 10.22038/IJBMS.2019.35125.8353. PMID: 32405356; PMCID: PMC7211351.
69. Wang Z, He C, Shi JS. Natural Products for the Treatment of Neurodegenerative Diseases. Curr Med Chem. 2020;27(34):5790-5828. doi: 10.2174/0929867326666190527120614.
70. Wang Z, Yang Y, Liu M, Wei Y, Liu J, Pei H, Li H. Rhizoma Coptidis for Alzheimer's Disease and Vascular Dementia: A Literature Review. Curr Vasc Pharmacol. 2020;18(4):358-368. doi: 10.2174/1570161117666190710151545.
71. Yadav SK, Ir R, Jeewon R, Doble M, Hyde KD, Kaliappan I, Jeyaraman R, Reddi RN, Krishnan J, Li M, Durairajan SSK. A Mechanistic Review on Medicinal Mushrooms-Derived Bioactive Compounds: Potential Mycotherapy Candidates for Alleviating Neurological Disorders. Planta Med. 2020 Nov;86(16):1161-1175. doi: 10.1055/a-1177-4834. Epub 2020 Jul 14. PMID: 32663897.
72. Yan Y, Yang H, Xie Y, Ding Y, Kong D, Yu H. Research Progress on Alzheimer's Disease and Resveratrol. Neurochem Res. 2020 May;45(5):989-1006. doi: 10.1007/s11064-020-03007-0.
73. Yang W, Ip SP, Liu L, Xian YF, Lin ZX. Uncaria rhynchophylla and its Major Constituents on Central Nervous System: A Review on Their Pharmacological Actions. Curr Vasc Pharmacol. 2020;18(4):346-357. doi: 10.2174/1570161117666190704092841.
74. Yao YY, Ling E-A, Lu D. Microglia mediated neuroinflammation - signaling regulation and therapeutic considerations with special reference to some natural compounds. Histol Histopathol 2020 Jul 14;18239. doi: 10.14670/HH-18-239.
75. Zhang F, Zhong RJ, Cheng C, Li S, Le WD. New therapeutics beyond amyloid-beta and tau for the treatment of Alzheimer's disease. Acta Pharmacol Sin . 2020 Dec 2. doi: 10.1038/s41401-020-00565-5.

B. 2019 year

1. Afzal M, Redha A, AlHasan R. Anthocyanins Potentially Contribute to Defense against Alzheimer's Disease. Molecules. 2019 Nov 22;24(23):4255. doi: 10.3390/molecules24234255.
2. Ali F, Siddique YH. Bioavailability and Pharmaco-therapeutic Potential of Luteolin in Overcoming Alzheimer's Disease. CNS Neurol Disord Drug Targets. 2019;18(5):352-365. doi: 10.2174/1871527318666190319141835. PMID: 30892166.
3. Barbalace MC, Malaguti M, Giusti L, Lucacchini A, Hrelia S, Angeloni C. Anti-Inflammatory Activities of Marine Algae in Neurodegenerative Diseases. Int J Mol Sci. 2019 Jun 22;20(12):3061. doi: 10.3390/ijms20123061.
4. Browne D, McGuinness B, Woodside JV, McKay GJ. Vitamin E and Alzheimer's disease: what do we know so far? Clin Interv Aging. 2019 Jul 18;14:1303-1317. doi: 10.2147/CIA.S186760. PMID: 31409980; PMCID: PMC6645610.
5. Bulgakova SV, Treneva EV, Zakharova NO, Trukhanova IG. [Intestinal microbiota: relationship to age-associated diseases (review of literature).]. Klin Lab Diagn. 2019;64(4):250-256. Russian. doi:10.18821/0869-2084-2019-64-4-250-256. PMID: 31108040.
6. Chen D, Huang C, Chen Z. A review for the pharmacological effect of lycopene in central nervous system disorders. Biomed Pharmacother. 2019 Mar;111:791-801. doi: 10.1016/j.biopha.2018.12.151.
7. Cheng LH, Liu YW, Wu CC, Wang S, Tsai YC. Psychobiotics in mental health, neurodegenerative and neurodevelopmental disorders. J Food Drug Anal. 2019 Jul;27(3):632-648. doi: 10.1016/j.jfda.2019.01.002. Epub 2019 Feb 10. PMID: 31324280.
8. Cremonini AL, Caffa I, Cea M, Nencioni A, Odetti P, Monacelli F. Nutrients in the Prevention of Alzheimer's Disease. Oxid Med Cell Longev. 2019 Sep 4;2019:9874159.
9. Dhakal S, Kushairi N, Phan CW, Adhikari B, Sabaratnam V, Macreadie I. Dietary Polyphenols: A Multifactorial Strategy to Target Alzheimer's Disease. Int J Mol Sci. 2019 Oct 14;20(20):5090. doi: 10.3390/ijms20205090. PMID: 31615073; PMCID: PMC6834216.
10. Dos Santos SM, Romeiro CFR, Rodrigues CA, Cerqueira ARL, Monteiro MC. Mitochondrial Dysfunction and Alpha-Lipoic Acid: Beneficial or Harmful in Alzheimer's Disease? Oxid Med Cell Longev. 2019 Nov 30;2019:8409329. doi: 10.1155/2019/8409329.
11. Elsaid S, Kloiber S, Le Foll B. Effects of cannabidiol (CBD) in neuropsychiatric disorders: A review of pre-clinical and clinical findings. Prog Mol Biol Transl Sci. 2019;167:25-75. doi: 10.1016/bs.pmbts.2019.06.005.
12. Fernández MJF, Valero-Cases E, Rincon-Frutos L. Food Components with the Potential to be Used in the Therapeutic Approach of Mental Diseases. Curr Pharm Biotechnol. 2019;20(2):100-113. doi: 10.2174/1389201019666180925120657. PMID: 30255749.
13. Ghahsare AG, Nazifi ZS, Nazifi SMR. Structure-Bioactivity Relationship Study of Xanthene Derivatives: A Brief Review. Curr Org Synth. 2019;16(8):1071-1077. doi: 10.2174/1570179416666191017094908

© 2021. Scassellati C et al. Published online at http://www.aginganddisease.org/EN/10.14336/AD.2021.0122

14. Gomez-Eguilaz M, Ramon-Trapero JL, Perez-Martinez L, Blanco JR. El eje microbiota-intestino-cerebro y sus grandes proyecciones [The microbiota-gut-brain axis and its great projections]. Rev Neurol. 2019 Feb 1;68(3):111-117. Spanish. PMID: 30687918.
15. Hajialyani M, Hosein Farzaei M, Echeverría J, Nabavi SM, Uriarte E, Sobarzo-Sánchez E. Hesperidin as a Neuroprotective Agent: A Review of Animal and Clinical Evidence. Molecules. 2019 Feb 12;24(3):648. doi: 10.3390/molecules24030648. PMID: 30759833; PMCID: PMC6384806.
16. Husain I, Zameer S, Madaan T, Minhaj A, Ahmad W, Iqubaal A, Ali A, Najmi AK. Exploring the multifaceted neuroprotective actions of Emblica officinalis (Amla): a review. Metab Brain Dis. 2019 Aug;34(4):957-965. doi: 10.1007/s11011-019-00400-9.
17. Hussain G, Huang J, Rasul A, Anwar H, Imran A, Maqbool J, Razzaq A, Aziz N, Makhdoom EUH, Konuk M, Sun T. Putative Roles of Plant-Derived Tannins in Neurodegenerative and Neuropsychiatry Disorders: An Updated Review. Molecules. 2019 Jun 13;24(12):2213. doi: 10.3390/molecules24122213.
18. Iranshahy M, Javadi B. Diet therapy for the treatment of Alzheimer's disease in view of traditional Persian medicine: A review. Iran J Basic Med Sci. 2019 Oct;22(10):1102-1117. doi: 10.22038/ijbms.2019.36505.8694. PMID: 31998450; PMCID: PMC6885391.
19. Jha NK, Jha SK, Kar R, Nand P, Swati K, Goswami VK. Nuclear factor-kappa β as a therapeutic target for Alzheimer's disease. J Neurochem. 2019 Jul;150(2):113-137. doi: 10.1111/jnc.14687.
20. Jin X, Liu MY, Zhang DF, Zhong X, Du K, Qian P, Gao H, Wei MJ. Natural products as a potential modulator of microglial polarization in neurodegenerative diseases. Pharmacol Res. 2019 Jul;145:104253. doi: 10.1016/j.phrs.2019.104253.
21. Kim Thu D, Vui DT, Ngoc Huyen NT, Duyen DK, Thanh Tung B. The use of Huperzia species for the treatment of Alzheimer's disease. J Basic Clin Physiol Pharmacol. 2019.
22. Lakey-Beitia J, Kumar D J, Hegde ML, Rao KS. Carotenoids as Novel Therapeutic Molecules Against Neurodegenerative Disorders: Chemistry and Molecular Docking Analysis. Int J Mol Sci. 2019 Nov 7;20(22):5553. doi: 10.3390/ijms20225553. PMID: 31703296; PMCID: PMC6888440.
23. Lin M, Xiang D, Chen X, Huo H. Role of Characteristic Components of <i>Humulus lupulus</i> in Promoting Human Health. J Agric Food Chem. 2019 Jul 31;67(30):8291-8302. doi: 10.1021/acs.jafc.9b03780. Epub 2019 Jul 18. PMID: 31287692.
24. Lloret A, Esteve D, Monllor P, Cervera-Ferri A, Lloret A. The Effectiveness of Vitamin E Treatment in Alzheimer's Disease. Int J Mol Sci. 2019 Feb 18;20(4):879. doi: 10.3390/ijms20040879.
25. Lu J, Ma Y, Wu J, Huang H, Wang X, Chen Z, Chen J, He H, Huang C. A review for the neuroprotective effects of andrographolide in the central nervous system. Biomed Pharmacother. 2019 Sep;117:109078. doi: 10.1016/j.biopha.2019.109078.
26. Martínez-Coria H, Mendoza-Rojas MX, Arrieta-Cruz I, López-Valdés HE. Preclinical Research of Dihydromyricetin for Brain Aging and Neurodegenerative Diseases. Front Pharmacol. 2019 Nov 11;10:1334. doi: 10.3389/fphar.2019.01334.
27. Pellegrini C, Fornai M, Antonioli L, Blandizzi C, Calderone V. Phytochemicals as Novel Therapeutic Strategies for NLRP3 Inflammasome-Related Neurological, Metabolic, and Inflammatory Diseases. Int J Mol Sci. 2019 Jun 13;20(12):2876. doi: 10.3390/ijms20122876.
28. Prasanth MI, Sivamaruthi BS, Chaiyasut C, Tencomnao T. A Review of the Role of Green Tea (Camellia sinensis) in Antiphotoaging, Stress Resistance, Neuroprotection, and Autophagy. Nutrients. 2019 Feb 23;11(2):474. doi: 10.3390/nu11020474. PMID: 30813433; PMCID: PMC6412948.
29. Rehman MU, Wali AF, Ahmad A, Shakeel S, Rasool S, Ali R, Rashid SM, Madkhali H, Ganaie MA, Khan R. Neuroprotective Strategies for Neurological Disorders by Natural Products: An update. Curr Neuropharmacol. 2019;17(3):247-267. doi: 10.2174/1570159X16666180911124605. PMID: 30207234; PMCID: PMC6425075.
30. Román GC, Jackson RE, Gadhia R, Román AN, Reis J. Mediterranean diet: The role of long-chain ω-3 fatty acids in fish; polyphenols in fruits, vegetables, cereals, coffee, tea, cacao and wine; probiotics and vitamins in prevention of stroke, age-related cognitive decline, and Alzheimer disease. Rev Neurol (Paris). 2019 Dec;175(10):724-741. doi: 10.1016/j.neurol.2019.08.005.
31. Salehi B, Venditti A, Sharifi-Rad M, Kręgiel D, Sharifi-Rad J, Durazzo A, Lucarini M, Santini A, Souto EB, Novellino E, Antolak H, Azzini E, Setzer WN, Martins N. The Therapeutic Potential of Apigenin. Int J Mol Sci. 2019 Mar 15;20(6):1305. doi: 10.3390/ijms20061305. PMID: 30875872; PMCID: PMC6472148.
32. Sasmita AO. Modification of the gut microbiome to combat neurodegeneration. Rev Neurosci. 2019 Nov 26;30(8):795-805. doi: 10.1515/revneuro-2019-0005. PMID: 31095511.
33. Sharma R, Kuca K, Nepovimova E, Kabra A, Rao MM, Prajapati PK. Traditional Ayurvedic and herbal remedies for Alzheimer's disease: from bench to bedside. Expert Rev Neurother. 2019 May;19(5):359-374. doi: 10.1080/14737175.2019.1596803.
34. Singh AK, Singh SK, Nandi MK, Mishra G, Maurya A, Rai A, Rai GK, Awasthi R, Sharma B, Kulkarni GT. Berberine: A Plant-derived Alkaloid with Therapeutic Potential to Combat Alzheimer's disease. Cent Nerv Syst Agents Med Chem. 2019;19(3):154-170. doi: 10.2174/1871524919666190820160053.

35. Singh AP, Singh R, Verma SS, Rai V, Kaschula CH, Maiti P, Gupta SC. Health benefits of resveratrol: Evidence from clinical studies. Med Res Rev. 2019 Sep;39(5):1851-1891. doi: 10.1002/med.21565. Epub 2019 Feb 11. PMID: 30741437.
36. Szczechowiak K, Diniz BS, Leszek J. Diet and Alzheimer's dementia -Nutritional approach to modulate inflammation. Pharmacol Biochem Behav. 2019 Sep;184:172743. doi: 10.1016/j.pbb.2019.172743. Epub 2019 Jul 26. PMID:31356838.
37. Tanaka M, Saito S, Inoue T, Satoh-Asahara N, Ihara M. Novel Therapeutic Potentials of Taxifolin for Amyloid-β-associated Neurodegenerative Diseases and Other Diseases: Recent Advances and Future Perspectives. Int J Mol Sci. 2019 Apr 30;20(9):2139. doi: 10.3390/ijms20092139.
38. Tinelli C, Di Pino A, Ficulle E, Marcelli S, Feligioni M. Hyperhomocysteinemia as a Risk Factor and Potential Nutraceutical Target for Certain Pathologies. Front Nutr. 2019 Apr 24;6:49. doi: 10.3389/fnut.2019.00049. PMID: 31069230; PMCID: PMC6491750.
39. Vlachos GS, Scarmeas N. Dietary interventions in mild cognitive impairment and dementia. Dialogues Clin Neurosci. 2019 Mar;21(1):69-82. doi: 10.31887/DCNS.2019.21.1/nscarmeas. PMID: 31607782; PMCID: PMC6780358.
40. Winter AN, Bickford PC. Anthocyanins and Their Metabolites as Therapeutic Agents for Neurodegenerative Disease. Antioxidants (Basel). 2019 Aug 22;8(9):333. doi: 10.3390/antiox8090333.
41. Zaplatic E, Bule M, Shah SZA, Uddin MS, Niaz K. Life Sci. 2019 May 1;224:109-119. doi: 10.1016/j.lfs.2019.03.055. Molecular mechanisms underlying protective role of quercetin in attenuating Alzheimer's disease. Life Sci. 2019 May 1;224:109-119. doi: 10.1016/j.lfs.2019.03.055.
42. Zhang J, Wu J, Liu F, Tong L, Chen Z, Chen J, He H, Xu R, Ma Y, Huang C. Neuroprotective effects of anthocyanins and its major component cyanidin-3-O-glucoside (C3G) in the central nervous system: An outlined review. Eur J Pharmacol. 2019.
43. Zhang X, Zhang S, Yang Y, Wang D, Gao H. Natural barrigenol-like triterpenoids: A comprehensive review of their contributions to medicinal chemistry. Phytochemistry. 2019 May;161:41-74. doi: 10.1016/j.phytochem.2019.01.017.
44. Zhao S, Zhang L, Yang C, Li Z, Rong S. Procyanidins and Alzheimer's Disease. Mol Neurobiol. 2019 Aug;56(8):5556-5567. doi: 10.1007/s12035-019-1469-6. Epub 2019 Jan 16. PMID: 30649713.

Supplementary Table 2. Summary of studies included in the meta-analyses.

References	markers	Models	N	Experiments	Treatments
[1]	NF-κB/p65 TLR4	Rats	36	Ischemia/Reperfusion (I/R) injury; Preconditioning with Ozone (O_3)	Rectal insufflations, 50μg/ml, 15 treatments, one a day.
[2]	NF-κB/p65 TLR4	Rats	30	Adenine diet and/or Ozone (O_3)	Rectal insufflations, 50μg/ml, 4 weeks, one a day.
[3]	NF-κB/p65	Rats	18	Adenine diet and/or Ozone (O_3)	Rectal insufflations, 50μg/ml, 4 weeks, one a day.
[4]	IL-17α	Mice	32	Dexamethasone injury;	Twice a week for 6 weeks.
[5]	NF-κB/p65 IL-17α NLRP3	Rats	30	Nephrectomy injury Preconditioning with Ozone (O_3)	Rectal insufflations, 50μg/ml, 10 weeks, one a day.
[6]	NF-κB/p65	Rats	40	NCLDH injury; Preconditioning with Ozone (O_3)	Intrathecal administration of 10, 20, or 30 μg/mL Ozone (O_3)
[7]	NF-κB/p65	Rats	54	Radiculoneuritis rat model; Preconditioning with Ozone (O_3)	Epidurally administration, 30 μg/ml, 3 days.
[8]	IL-17α NLRP3	Rats	18	Ischemia/Reperfusion (I/R) injury; Preconditioning with Ozone (O_3)	Rectal insufflations, 100μg/ml, 10 days, one a day.

© 2021. Scassellati C et al. Published online at http://www.aginganddisease.org/EN/10.14336/AD.2021.0122

[9]	IL-17α<br>NLRP3	Human	40 patients;<br>20 healthy subjects	Multiple sclerosis	Autohemotherapy method, 25 µg/ml, twice weekly for 6 months.

NF-κB/p65 (nuclear factor kappa-light-chain-enhancer of activated B cells); IL-17α (pro-inflammatory Interleukin 17), NLRP3 Inflammasome (NLR Family Pyrin Domain Containing 3); N= sample size

© 2021. Scassellati C et al. Published online at http://www.aginganddisease.org/EN/10.14336/AD.2021.0122

SUPPLEMENTARY DATA

References

[1] Xing et al. Ozone oxidative preconditioning protects the rat kidney from reperfusion injury via modulation of the TLR4-NF-kappaB pathway. Acta Cir Bras. 2015.
[2] Chen et al. Ozone therapy ameliorates tubulointerstitial inflammation by regulating TLR4 in adenine-induced CKD rats. Ren Fail. 2016.
[3] Yu et al. Ozone therapy could attenuate tubulointerstitial injury in adenine-induced CKD rats by mediating Nrf2 and NF-kappaB. Iran J Basic Med Sci. 2016.
[4] Fei et al. IL-17A Monoclonal Antibody Partly Reverses the Glucocorticoids Insensitivity in Mice Exposed to Ozonec. Inflammation. 2017
[5] Yu et al. The NLRP3 inflammasome is a potential target of ozone therapy aiming to ease chronic renal inflammation in chronic kidney disease. Int Immunopharmacol. 2017.
[6] Wang et al. Low-Concentration Oxygen/Ozone Treatment Attenuated Radiculitis and Mechanical Allodynia via PDE2A-cAMP/cGMP-NF-kappaB/p65 Signaling in Chronic Radiculitis Rats. Pain Res Manag. 2018.
[7] Wu et al. Therapeutic dosage of ozone inhibits autophagy and apoptosis of nerve roots in a chemically induced radiculoneuritis rat model. Eur Rev Med Pharmacol Sci. 2018.
[8] Wang et al. Ozone protects the rat lung from ischemia-reperfusion injury by attenuating NLRP3-mediated inflammation, enhancing Nrf2 antioxidant activity and inhibiting apoptosis. Eur J Pharmacol. 2018.
[9] Izadi et al. Changes in Th17 cells frequency and function after ozone therapy used to treat multiple sclerosis patients. Mult Scler Relat Disord. 2020

© 2021. Scassellati C et al. Published online at http://www.aginganddisease.org/EN/10.14336/AD.2021.0122
